# Machine Learning Based Clinical Decision Support System for Early COVID-19 Mortality Prediction

**DOI:** 10.3389/fpubh.2021.626697

**Published:** 2021-05-12

**Authors:** Akshaya Karthikeyan, Akshit Garg, P. K. Vinod, U. Deva Priyakumar

**Affiliations:** Center for Computational Natural Sciences and Bioinformatics, International Institute of Information Technology, Hyderabad, India

**Keywords:** coronavirus disease 2019, prognosis, mortality, biomarkers, machine learning

## Abstract

The coronavirus disease 2019 (COVID-19), caused by the virus SARS-CoV-2, is an acute respiratory disease that has been classified as a pandemic by the World Health Organization (WHO). The sudden spike in the number of infections and high mortality rates have put immense pressure on the public healthcare systems. Hence, it is crucial to identify the key factors for mortality prediction to optimize patient treatment strategy. Different routine blood test results are widely available compared to other forms of data like X-rays, CT-scans, and ultrasounds for mortality prediction. This study proposes machine learning (ML) methods based on blood tests data to predict COVID-19 mortality risk. A powerful combination of five features: neutrophils, lymphocytes, lactate dehydrogenase (LDH), high-sensitivity C-reactive protein (hs-CRP), and age helps to predict mortality with 96% accuracy. Various ML models (neural networks, logistic regression, XGBoost, random forests, SVM, and decision trees) have been trained and performance compared to determine the model that achieves consistently high accuracy across the days that span the disease. The best performing method using XGBoost feature importance and neural network classification, predicts with an accuracy of 90% as early as 16 days before the outcome. Robust testing with three cases based on days to outcome confirms the strong predictive performance and practicality of the proposed model. A detailed analysis and identification of trends was performed using these key biomarkers to provide useful insights for intuitive application. This study provide solutions that would help accelerate the decision-making process in healthcare systems for focused medical treatments in an accurate, early, and reliable manner.

## Introduction

The outbreak of the novel coronavirus disease 2019 (COVID-19) caused by the severe acute respiratory syndrome coronavirus 2 (SARS-CoV-2) began in Wuhan, China in December 2019. Since then, it has rapidly spread around the world. As of April 24, 2021, WHO reported a total of about 144 million confirmed cases and more than 3 million deaths worldwide. At this stage of the pandemic, most estimates of fatality ratios have been based on cases detected through surveillance and calculated using crude methods, giving rise to widely variable estimates of case fatality rate by country. The sudden rise in cases has put immense pressure on the healthcare systems due to limited resources. Identification of the key bio-markers of mortality is crucial because it helps in understanding the relative risk of death among patients and therefore guides policy decisions regarding scarce medical resource allocation during the ongoing COVID-19 pandemic.

Symptoms of COVID-19 are very similar to the common flu that include fever, cough and nasal congestion (1). As the pandemic spread, other symptoms such as loss of taste and smell (anosmia) have emerged (2, 3). Severe cases can lead to serious respiratory disease and pneumonia. Those most at risk are the elderly and people with underlying medical issues/comorbidities, such as cardiovascular diseases and diabetes (4–6). As the disease spreads around the world, more symptoms and features that affect patient mortality are being realized. Having such a large set of features that are affected by the disease makes it hard to understand which ones have a greater impact on patient mortality. Machine learning can aid by analyzing large sets of data to find patterns quickly and providing models that assess risk factors accurately.

Several studies have applied ML algorithms for detecting and diagnosing COVID-19 infection in patients (3, 7–12). These provide a scope for faster screening of patients during pandemic and help in overcoming challenges in performing Reverse Transcription Polymerase Chain Reaction (RT-PCR) at the population-scale. Most ML-driven COVID-19 studies are based on Chest X-rays or CT-scans (7). Such data is difficult to obtain in a low resource setting (13, 14). Thus, there is a need to develop model based on alternative data obtained using easily accessible and inexpensive tests. The detection of COVID-19 based on cough samples has shown promise (9). However, a scalable solutions are required to predict severity of COVID-19 infection. Routine blood tests have shown promise in severity prediction.

Different ML models have been proposed to predict risk of developing severe complications and mortality (15–27). This is important since there are limited resources compared to the increasing number of COVID-19 patients. The resource allocation and distribution among patients depending on their prognosis is an important issue. Wang et al. (28) proposed two different models based on Clinical and Laboratory features to predict mortality of COVID-19 patients. The Clinical model developed with Age, history of hypertension and coronary heart disease showed AUC of 0.83 (95% CI, 0.68–0.93) on the validation cohort. The laboratory model developed with age, high-sensitivity C-reactive protein (hs-CRP), peripheral capillary oxygen saturation (SpO2), neutrophil and lymphocyte count, D-dimer, aspartate aminotransferase (AST) and glomerular filtration rate (GFR) had better discriminatory power with AUC of 0.88 (95% CI, 0.75–0.96) on the validation cohort. The Validation cohort consisted of 44 COVID-19 patients of which 14 died and 30 survived. XGBoost and backward step-down selection were used for feature selection followed by a multivariate logistic regression for the classification. The clinical model can prove to be useful given the ease of data collection of all the three features. Shang et al. (29) established a scoring system of COVID-19 (CSS) to split patients into low-risk and high-risk groups. Here, high-risk group patients would have significantly higher chances of death than those of the low-risk group. Multivariable analysis and coefficients of lasso binary logistic regression were used to do feature analysis and to establish a prediction model. Eight different variables including age and blood parameters were used to generate a model which showed good discriminative power with an AUC of 0.938 (95% Cl, 0.902–0.973) on the independent validation cohort. Xie et al. (30) identified SpO2, Lymphocyte Count, Age, and Lactate dehydrogenase (LDH) as a set of important features to generate a mortality prediction model. They used multivariable logistic regression for the classification task which gave an AUC of 0.98 on the independent validation set. Using this they established a nomogram to generate probability of mortality. Jimenez-Solem et al. (24) used COVID-19 data from Denmark and UK to build mortality prediction models which performed with AUC of 0.906 at diagnosis, 0.818 at hospital admission, and 0.721 at ICU admission. Common risk factors, included age, body mass index, and hypertension. Bolourani et al. (22) developed ML models to predict respiratory failure within 48 h of patient admission for COVID-19. XGBoost model had the highest mean accuracy of 0.919 and AUC of 0.77. The most influential variables included the type of oxygen delivery used in the emergency department, age, Emergency Severity Index level, respiratory rate, serum lactate, and demographic characteristics. Most studies have not presented the consistency of their results with different days to outcome. This analysis is necessary to assess the model's performance and its reliable application in real scenarios where a patient may be in any stage in the duration of the disease.

An interpretable mortality prediction model for COVID-19 patients was proposed by Yan et al. (31) where they analyzed blood samples of 485 patients from Wuhan, China, and developed an XGBoost based solution. The proposed clinically operable single tree XGBoost model used three crucial features- LDH, lymphocytes, and hs-CRP. The decision rules with the three features along with their threshold were devised recursively. This provided a interpretable machine learning solution along with an accuracy of atleast 90% for all days. Yan et al. analyzed their model consistency and showed 94% accuracy at 7 days before day of outcome. However, it's possible that their results are biased (32). The method used to determine consistency was skewed toward the high number of samples near the day of outcome. The F1-score starts from 0.97 on day 1 and drops to 0.68 on day 17 from the outcome, showing inconsistency. Moreover, the unbalanced test set brings unreliability into the results. It is desirable to predict accurately during the initial days of infection, which helps to devise treatment strategies early.

In this study, we propose a machine learning pipeline to overcome these shortcomings in mortality prediction and to improve the performance. We have analyzed the dataset provided by Yan et al., which contains the biomarkers derived from blood tests, for developing our models. We created a solution using XGBoost feature importance and neural net (NN) classification. We have performed feature selection to minimize the numbers of features that can be used for developing risk stratification models. A large number of features for a small cohort may sometimes lead to overfitting. A strong combination of five features was selected as the key biomarkers for prediction. The neural net provides a high predictive performance (96% accuracy), while the XGBoost feature importance graph adds interpretability to the model. Analysis of the features' graphs showing clear trends in progression provide additional insights into the features as well. Various algorithms and robust testing were implemented to establish a strong confidence in the model. Our model proves to be extremely accurate and consistent through the days spanning a patient's disease. This would help in faster diagnosis with fewer number of features and higher confidence. Healthcare systems can use the model to optimize treatment strategy by focused utilization of resources.

## Methodology

### Dataset and Preprocessing

All data for feature analysis, training and testing were taken from (31). This dataset includes 2,779 electronic records of validated or suspected COVID-19 patients from Tongji Hospital in Wuhan, China. The initial dataset comprised of the time series data of 375 COVID-19 patients with 74 biomarkers along with data sample time, admission time, discharge time and outcome (survived or dead). Yan et al. (31) used only data of the final samples of each patient for training and testing. In our study, we have considered the samples from all the days of each patient for training and testing. For each patient, there were multiple rows representing readings taken on different days. Some days also had multiple readings taken at different times. All such rows representing same day readings of a patient were combined together to create a single data point for each unique day of the patient. In cases where there existed features with multiple recordings taken in a single day at different times, the readings which was taken the earliest in that particular day is considered for generating the combined single data point. This is because we require the model to learn the features that predict mortality at the earliest. Features which had missing values in more than 70% of the instances were dropped and were not used for further analysis. A new column “Number of days till Outcome” is added to signify how many days are left for a sample to reach the day of outcome. This is calculated by subtracting the day of reading from the day of discharge/death. After data generation and processing, our dataset had 201 patients who survived and 169 patients who died. The features were analyzed with respect to the two classes.

Missing values in the training set were imputed using K-Nearest Neighbor algorithm. The value for imputation was calculated by averaging out the values of 10 nearest neighbors weighted with respect to inverse of Euclidean distance. For normalizing the training data Min-Max scaling was used. Min-Max scaling was used since most features don't fit a normal distribution and to ensure that the structure of the data-points in each feature remained intact.

### Data Splitting for Unique Patient Segregation

**Figure 1:**
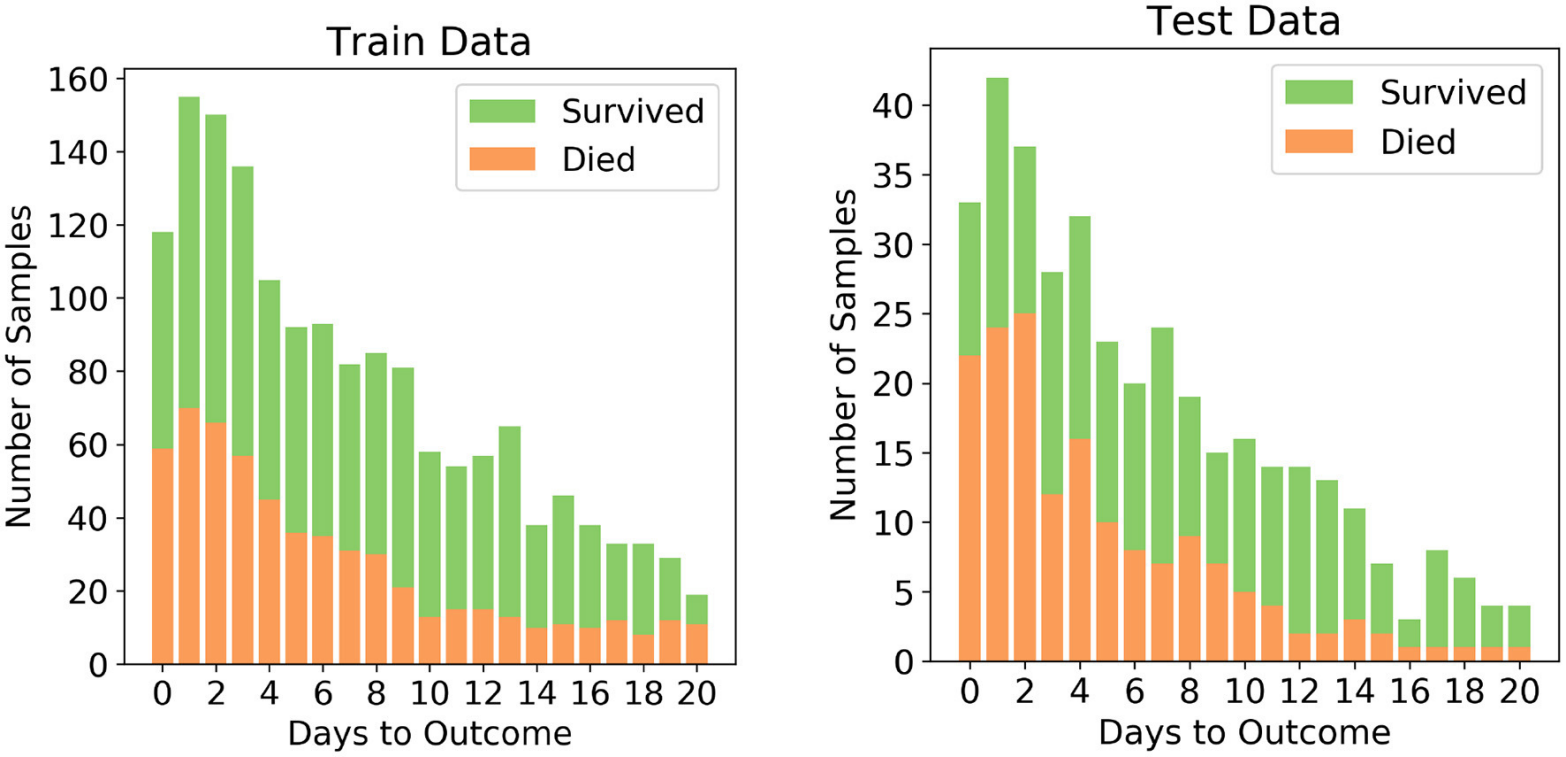
Distribution of the two classes in the train and test sets after splitting.

The dataset after pre-processing consists of 1,766 datapoints corresponding to 370 patients, out of whom 54.3% recovered and 45.7% succumbed to COVID19. The number of datapoints for each of the patients range from 1 to 12 that were collected during different days before one of the two outcomes. To ensure exclusivity of patients in the training and testing sets, 80% of the patients were randomly chosen for the training set and remaining 20% of the patients were chosen for the testing set. Unique patient segregation is important since including datapoints from a single patient in the training and test sets may lead to bias. Such a unique 80:20 split gave us a training set comprising of 1,418 data points and testing set comprising of 348 data points. The class distribution across the days to outcome is shown in Figure 1. The classes are spread across all the days in good ratios and are comparable. Since our aim is to develop mortality prediction model that is independent on the days to outcome, all the readings of the patients are considered as unique data-points for further examination.

In this study, results with and without K-Nearest Neighbor (KNN) imputation are compared. KNN algorithm is useful for matching a data-point with its closest *k* neighbors in a multi-dimensional space. *Imputed test set:* Each data-point in the test set was imputed using the values corresponding to the selected five features from their ten nearest neighbors in the train set. The resulting test set contained 213 samples belonging to 71 patients. The dead/total patient ratio of 0.563 shows that there is a good representation of both classes in the test set. This ensures that the results are reliable for both classes. *Non-imputed test set:* To assess the performance of the algorithms when the test set has no synthesized values, we dropped the rows that had missing values in any one of the five features. The resulting test set contained 115 samples belonging to 65 patients with a dead/total patient ratio of 0.513.

### Machine Learning Pipeline

**Figure 2:**
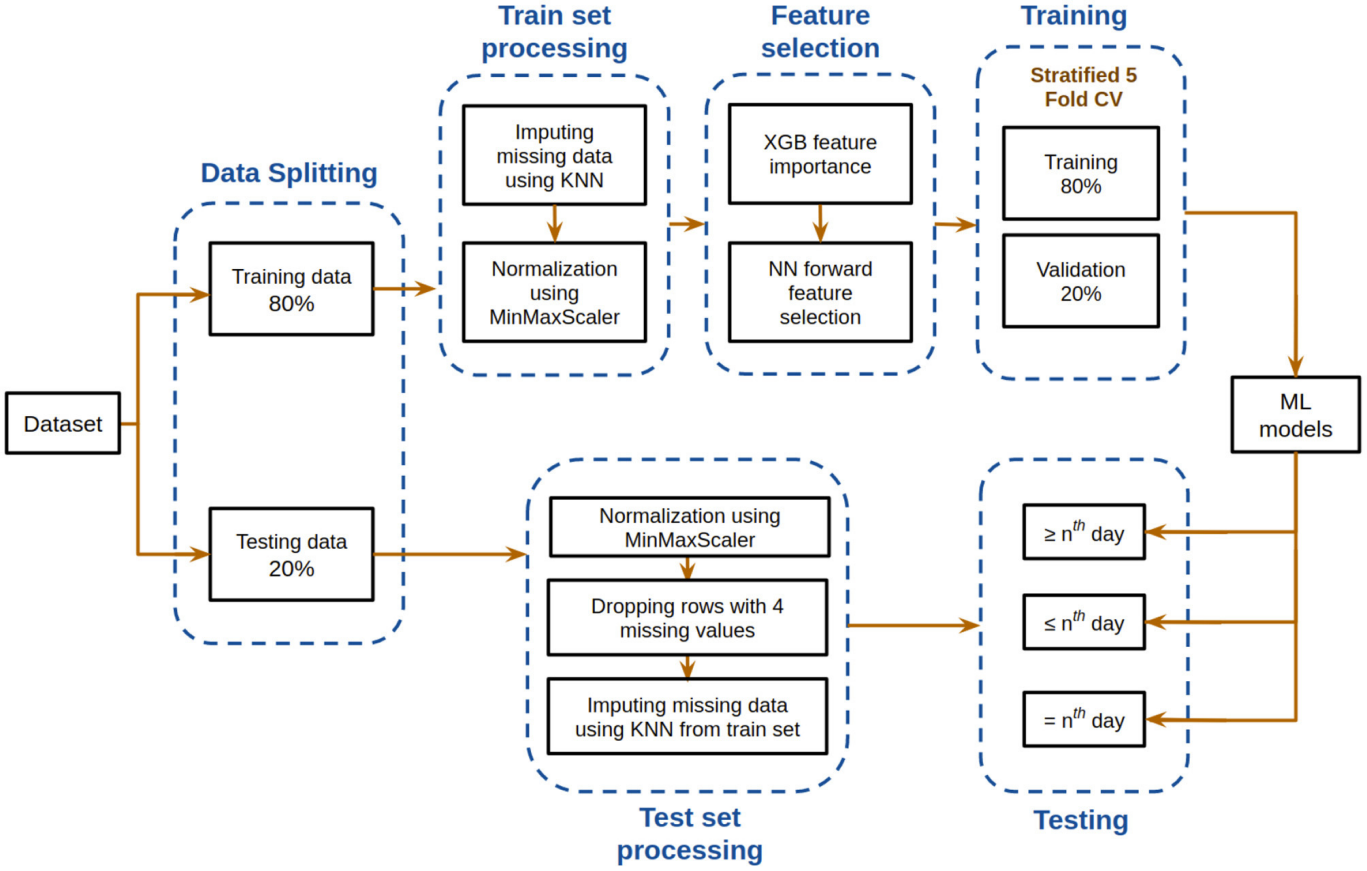
Flowchart depicting the model development pipeline used in this study.

Figure 2 depicts the overall pipeline used in this study for performing the mortality prediction task. All the models have been trained on samples from all days from this dataset for mortality prediction irrespective of the number of days to outcome. Following data preprocessing, XGBoost classifier was used to obtain feature importance, and a neural network was used for feature selection. The optimum combination of features thus obtained was then used to train various supervised machine learning classification models. The trained models were then tested based on three different ways, with each having its own strengths. Five-fold cross validation was utilized to assess the predictive ability and statistical significance of the models. Assessment of the developed models was done based on different metrics whose mean and standard deviation are reported below. Further detailed account of the step-by-step procedure is presented below.

### Evaluation Metrics

The predictive performance of the supervised models was assessed using the following metrics. Here, TP, TN, FP, and FN stand for true positive, true negative, false positive, and false negative rates, respectively).

*ROC-AUC:* AUC stands for “Area under Receiver Operating Characteristic curve” (ROC curve in short). It provides an aggregate measure of performance across all possible classification thresholds. The ROC curve plots two parameters:

• True Positive Rate

$$TPR=\frac{TP}{TP+FN}$$

• False Positive Rate

$$FPR=\frac{FP}{FP+TN}$$

AUC measures the entire two-dimensional area under the ROC curve starting from (0,0) and ending at (1,1).

*Accuracy:* Accuracy is an important metric for classification models. In this study, the test dataset is not unbalanced, hence this will give a good idea about the model's predictive performance.

$$Accuracy=\frac{TP+TN}{TP+TN+FP+FN}$$

*F1 Score:* F1 score is the harmonic mean of precision and recall.

$$F1score=\frac{2\times Precision\times Recall}{Precision+Recall}$$

where,

$$Precision=\frac{TP}{TP+FP}$$

and,

$$Recall=\frac{TP}{TP+FN}$$

### XGBoost Feature Importance

To evaluate the biomarkers that have the most influence on the outcome, XGBoost was used to get the relative importances. XGBoost is a powerful machine learning algorithm that estimates features that are the most discriminative of model outcomes (33). The final importance of a feature is calculated using the mean of its importances across all the trees. The importance of a feature over a tree is determined by the number of times the feature is used to split the tree, which is weighted as the square of the improvement made by the split in the model performance (34) The average importances of the features were found through 100 iterations of randomly selecting 80% of the samples in the training set. The parameter settings for XGBoost of maximum depth was set to 3, learning rate was set to 0.2 and regularization parameter α was set to 5 with logistic regression as the objective. All the other parameters carry their default values.

### NN Feature Selection

**Figure 3:**
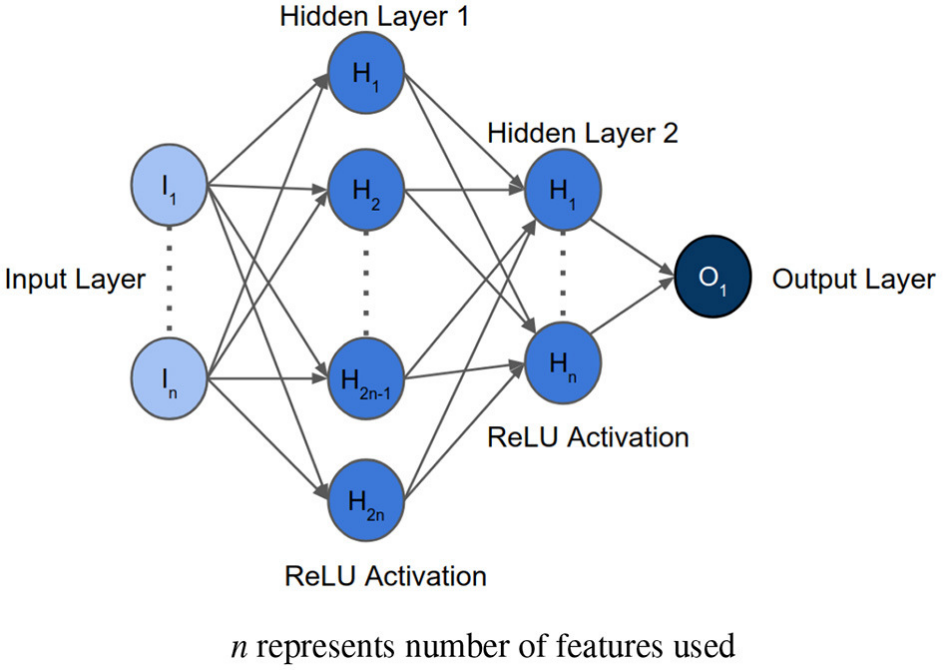
Architecture of the neural network implemented for feature selection, where *n* represents the number of features to be analyzed.

After determining the order of importance, neural network was used to find the optimal number of features required for mortality prediction. NNs can learn complex relations between the features. The input layer had the same number of neurons as the number of features to be analyzed. The architecture included two hidden layers with ReLU activation. The first hidden layer had double the number of neurons as the input layer, and the second hidden layer had equal number of neurons as the input layer. The Figure 3 shows the architecture of the neural network. Binary Cross Entropy with Logits was the loss function. Adam optimizer with learning rate 0.001 and Reduce On Plateau scheduler with patience as 5 was utilized to update the weights and learning rate. Theoretically, shallow NN is capable of performing as good as or better than logistic regression, given the NN is not overfitting on the training set (35). We used Early Stopping and Learning Rate Decay to prevent overfitting. The average AUC of each set during feature selection was calculated using stratified 5-fold cross validation. The performance of each set of features was then compared to select the optimum one.

### Training

To compare the performances of various machine learning algorithms, we tried six different models as listed below. The most optimum feature set was used as inputs to these models. GridCV with Stratified 5-fold cross validation was used for extensive hyper-parameter tuning of Logistic Regression, Random Forests, XGBoost, Support Vector Machine, and Decision Trees.

1. Neural Network: For training, a similar Neural Network as the one used for feature selection was developed for predicting mortality. The number of neurons in the input layer was equal to the optimal number of features as determined through feature selection. The first and second hidden layers thus have double and equal number of neurons as the input layer, respectively. The learning rate was set as 0.00001 and patience of the scheduler was set as 50 to ensure good fitting on the training data. The cutoff for classification which was evaluated with respect to the validation set and F1-Score was used as the metric for performance comparison. In case multiple cutoffs gave the same F1-Score, the cutoff closest to 0.5 was chosen.
2. Logistic Regression: Logistic Regression is an interpretable model that performs well on simple data that is linearly separable (36, 37). It was trained with “liblinear” solver due to small dataset size, L1 penalty, tolerance for stopping criteria set as 0.0001, inverse of regularization strength *C* set as 10 and intercept scaling set as 1.
3. Random Forests (RF): RF is a robust, tree based technique useful for handling missing data and outliers (38). It maintains its accuracy despite data having small size (39). It was trained with gini criterion, maximum depth set as 9, minimum number of samples at a leaf node set as 1, and number of trees set as 90.
4. XGBoost: XGBoost was trained with objective set as logistic regression, gamma set as 0, learning rate set as 0.2, maximum delta step set as 0, maximum depth set as 4, minimum sum of instance weight needed in a child set as 0, L2 regularization parameter lambda set as 7 and subsample set as 1.
5. Support Vector Machine (SVM): SVM is a machine learning technique that has excellent generalization capacity and also useful for small data size (40). On comparing four types of kernels [i.e., linear, polynomial (“poly”), radial basis function (RBF), and sigmoid], it was observed that the “poly” kernel performed best. Hence, the SVM model was trained with “poly” kernel, degree set as 3, gamma set as scale, regularization parameter *C* as 5, and maximum iterations set as 500,000.
6. Decision Trees: Decision Trees predict the dependent variable's values by learning simple decision rules inferred from the data (41, 42). It was trained with criterion set as “entropy,” maximum depth set as 9, minimum number of samples required to be at a leaf node set as 9, minimum number of samples required to split an internal node set as 2, and splitter set as “random.”

### Testing

Stratified five-fold cross validation was used to acquire five models that were trained and validated on different folds (43). Due to a small dataset size, five-fold cross validation was chosen for sufficient variation such that the underlying distribution is represented. These models were then tested on the test set, and the results were averaged to determine model predictive performance.

*Test set processing:* The test set was first normalized using Min-Max scaling that was fit on the train set. Some of the rows had missing values, so we processed them in two ways as follows:

1. Rows missing values for any four or more of the five selected features were eliminated. The missing values of each sample in the resulting test set were imputed with the average of the values of its corresponding 10 nearest neighbors in the train set using KNN, where nearest neighbors were found with respect to the selected five features only. The nearest neighbors were determined through the inverse of Euclidean distance between the data-points.
2. All the rows missing any one of the five values were dropped. This set has no imputation, and only includes the rows that have had all the values. This produces a test set which can simulate 100% real life testing scenario.

*Testing on three cases:* For realizing the true predictive performance and its consistency, the models were tested using three cases. Each of the following three cases have their own significance:

1. Case ≤ n: If only *n* or less days are left till outcome Test samples that had the value of “Number of days till Outcome” as greater than *nth* day were dropped. Then, testing was done on the cumulation of the rest of the samples.
2. Case ≥n: For *n* or more days in advance Test samples that had the value of “Number of days till Outcome” as lesser than *nth* day were dropped. Then, testing was done on the cumulation of the rest of the samples.
3. Case = n: On exactly *n* days before the outcome Test samples that had the value of “Number of days till Outcome” equal to *nth* day were chosen.

## Results

### Identification of Key Biomarkers

Given, especially, the severity and rapid spike in COVID-19 infections and resulting fatalities, a large number of lab-tests is required to assess patients' medical conditions. Feature selection by which the most important and crucial biomarkers that aid in risk assessment are identified, is an important exercise because acquiring fewer lab tests means faster and efficient decision-making processes.

*XGBoost feature importance*: The relative importance of available features were determined using the gradient boosting algorithm, XGBoost. It was observed that the top four features are neutrophils (%), lymphocyte (%), LDH and hs-CRP in the given order. Supplementary Figure 1 shows the order of relative importance of all the features originally considered. The list of features was sorted in the descending order of importance. “Age” was then added to the top of the feature importance list owing to its extreme ease of data collection and studies (4–6) showing that its an important factor in determining disease progression of COVID-19 patients. The distribution of the five selected features with respect to both the classes- survived and dead is given in Supplementary Figure 2.

*NN feature selection*: The AUC scores of each set of features during forward selection using a neural network was plotted. The aim of this exercise was to maximize AUC score and minimize the number of features selected for model development. It was observed that the mean AUC score at five features was 0.95. Adding the sixth feature did not increase the AUC significantly. Observed AUC with respect to the number of features selected for modeling is given in Supplementary Figure 3. It was also noticed that dropping “age” feature hampered the performance of the model, whereas adding “gender” did not improve the model performance. Hence, the features selected for this study are age, neutrophils (%), hs-CRP, lymphocyte (%), and LDH. These selected features are also statistically significant (with *p*-value <0.001) checked using ANOVA *F*-test.

### Predictive Ability of the Model

**Figure 4:**
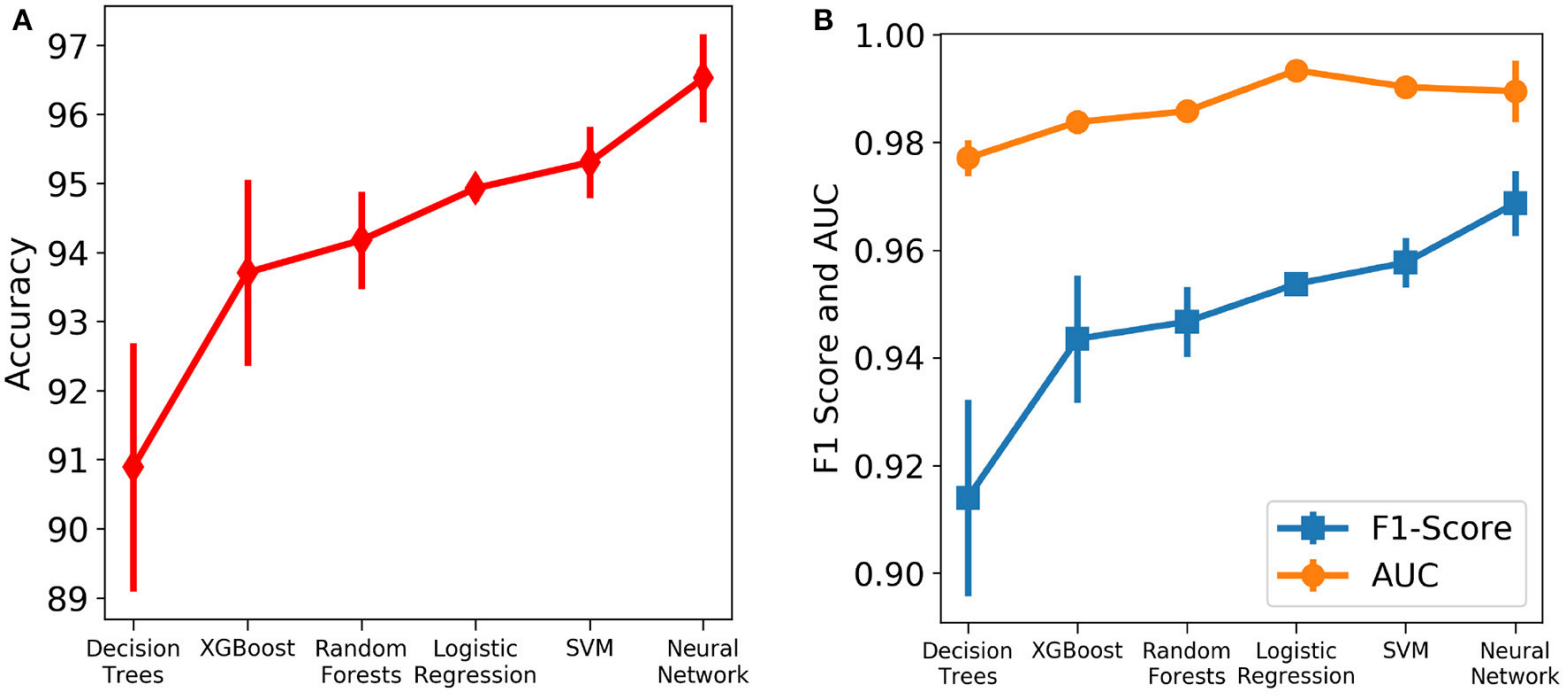
Comparison of the performance of different machine learning algorithms assessed using different metrics. The vertical lines denote the standard deviations. **(A)** Accuracy. **(B)** AUC and F1 score.

Six different algorithms, namely neural net, SVM, logistic regression, random forests, XGBoost and decision trees, were used to develop the classification models. Initially, each algorithm after stratified five-fold cross validation was tested on the test set, which included samples from all days. Figure 4 shows the accuracy, F1 Score and AUC of all the developed models using the six different methods. Our aim is to choose the most accurate model that is highly capable of distinguishing the two classes. It was observed that the best model is the neural net as it performed better than rest in terms of accuracy (96.53 ± 0.64%) and F1 score (0.969 ± 0.006; Supplementary Table 1). Supplementary Figure 4 shows its loss curve, depicting a good fit. It has a high AUC score (0.989 ± 0.006), showing good distinguishing capacity for the features of both classes. Hence, the results and discussions presented in the rest of the manuscript will be based on those obtained using the neural network unless otherwise mentioned.

**Figure 5:**
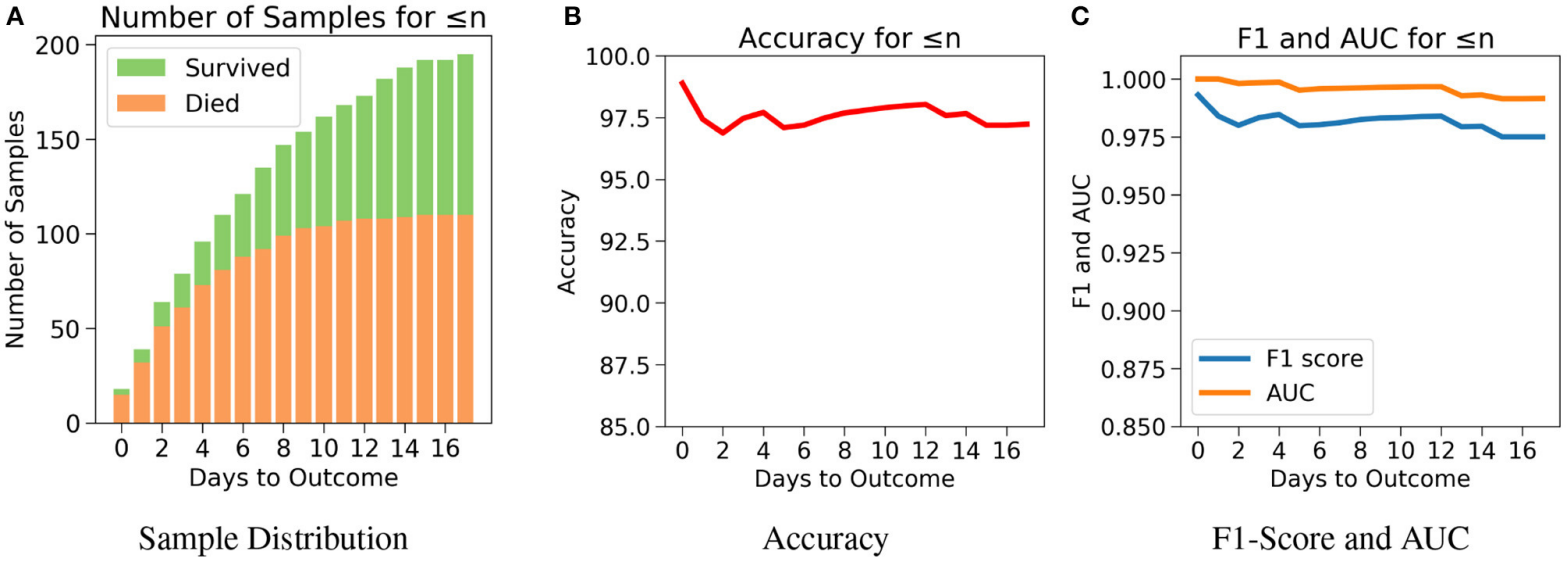
The performance of neural net on the test data using case 1: Number of days to outcome less than or equal to *n*. **(A)** The class-wise distribution of the cumulated data-points (≤ *nth* day) for all samples in the imputed test set. **(B)** Accuracy of the model evaluated for different days to outcome. **(C)** F1-score and AUC of the model evaluated for different days to outcome.

The robustness and applicability of the model at different settings were further tested by considering three different cases (Cases 1, 2, and 3). The first case investigates the performance of the model when the test set consists of data only if *n* or less number of days are left till the day of outcome (Figure 5A). Testing was done with respect to different values of *n*. From Figure 5B, it was observed that the accuracy is consistently high for *n* up to 17 days if only *n* or less days are left till the outcome. For the value of range of *n* between 0 and 17 days, the accuracy values were in a close range of 97.1–99.0% indicating the high predictive nature of the model. The number of samples after day 17 is relatively very few to affect this accuracy at later days, hence all the analysis have been done up to only 17 days. From Figure 5C, we observe high and consistent AUC scores (best = 1 and worst = 0.99) and F1 scores (best = 0.99 and worst = 0.97) across various days. This shows that the model performs consistently with an accuracy of at least 97% if any number of days are left till the outcome.

**Figure 6:**
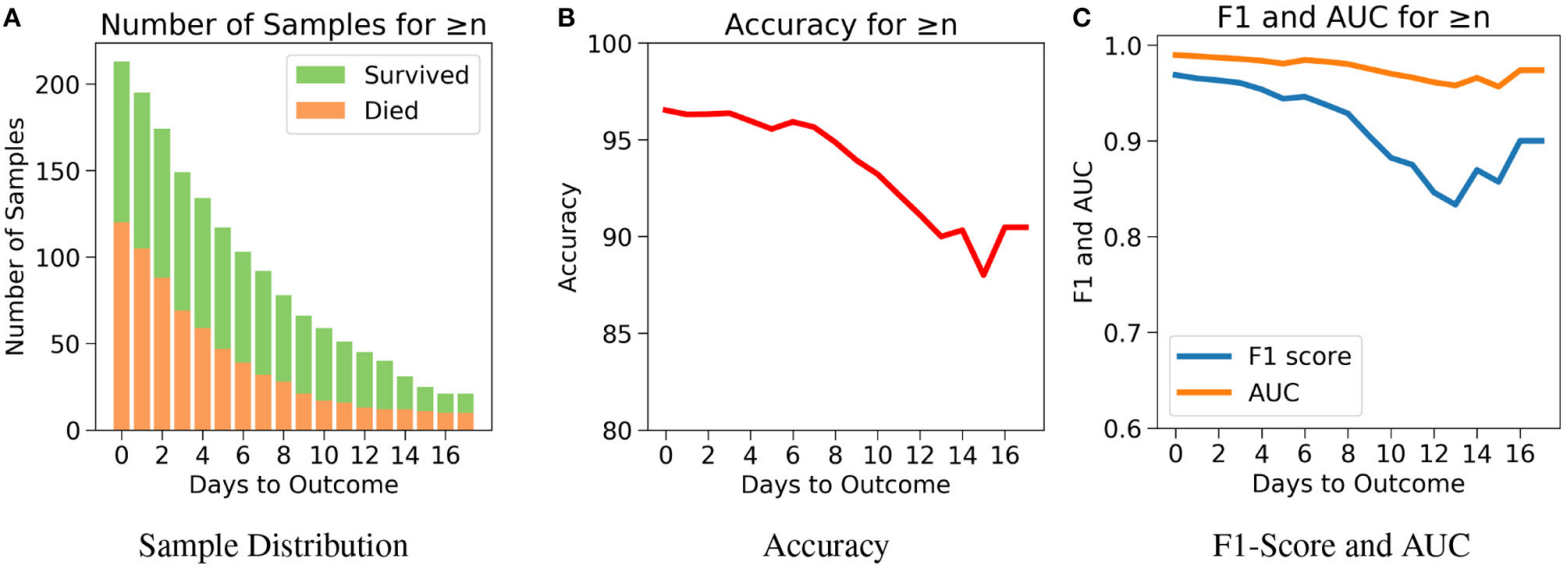
The performance of neural net on the test data using case 2: Number of days to outcome greater than or equal to *n*. **(A)** The class-wise distribution of the cumulated data-points (≤ *nth* day) for all samples in the imputed test set. **(B)** Accuracy of the model evaluated for different days to outcome. **(C)** F1-score and AUC of the model evaluated for different days to outcome.

The drawback of Case 1 is that it is possible for the samples nearer to the outcome to dominate the results since there are more samples nearer to the outcome. Case 2, which examines the performance of the model for *n* or more number of days in advance, was considered to address this drawback. One does not know when the day of outcome is going to be; hence it is important to analyze the model's performance with respect to any day before the day of outcome. This would also help to assess the performance of the model over the span of the disease. Since the number of samples decreases with respect to the number of days before outcome, every cumulation can only be dominated by the samples of the day closest to the outcome (Figure 6A). Hence, this gives a more accurate overview. Figure 6B agrees with our intuition, that it gets harder to predict the outcome as we go farther from the day of outcome. Nevertheless, the model starts with a high accuracy of 96.5% at the day of outcome and stays quite consistent. The lowest accuracy of 88% was observed when the model predicts 15 or more days in advance. From Figure 6C, a similar consistency was observed with the AUC and F1 scores. The AUC starts with 0.99 at the day of outcome and reaches its lowest point on day 15 with 0.96. F1 scores start with 0.97 and reach its lowest point 0.84 on day 13. However the model predicts with a high accuracy of 95.7% when *n* ≥ 1 week indicating strong performance.

**Figure 7:**
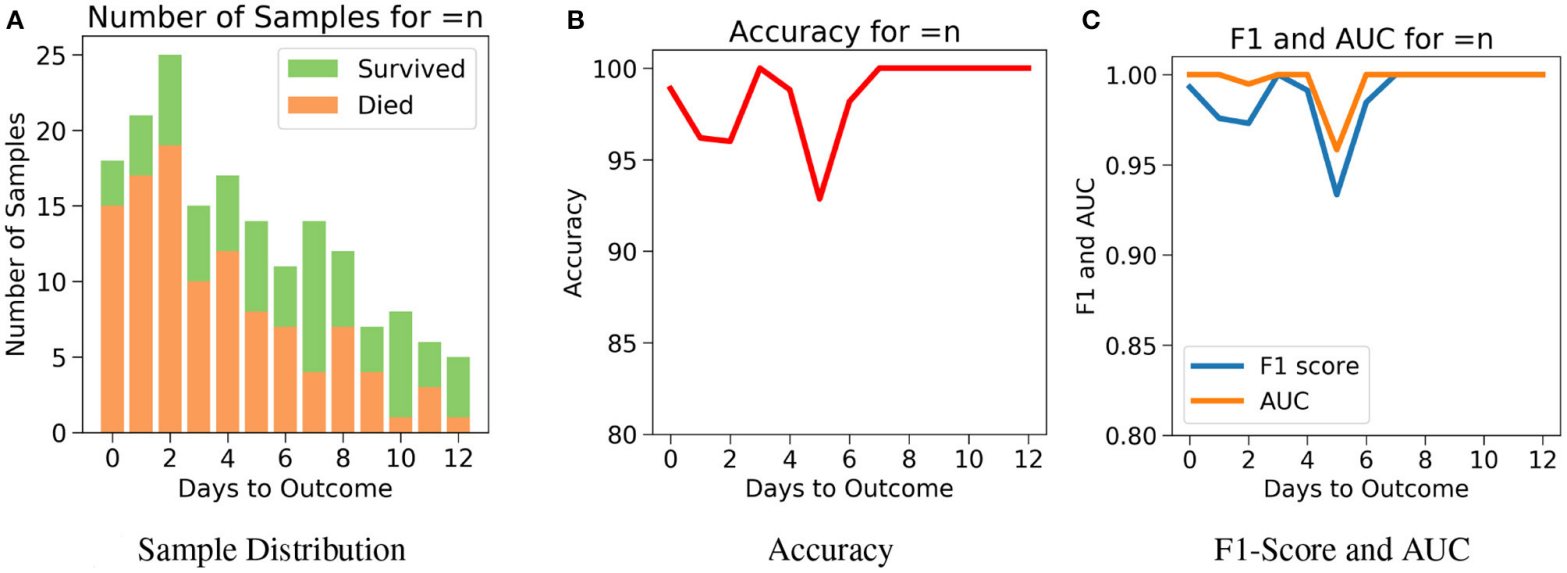
The performance of neural net on the test data using case 3: Number of days to outcome equal to *n*. **(A)** The class-wise distribution of the cumulated data-points (≤ *nth* day) for all samples in the imputed test set. **(B)** Accuracy of the model evaluated for different days to outcome. **(C)** F1-score and AUC of the model evaluated for different days to outcome.

Next, for even more rigorous testing, we introduce Case 3 which assesses the performance of the model on exactly the *nth* day before the day of outcome (Figure 7A). Here, no sample from any other day can influence the results. Even though the accuracy fluctuates through the days, Figure 7B shows that the model predicts the outcome with an accuracy of 98.9% on the day of outcome and reaches its lowest value of 92.85% on day 5. The AUC and F1 scores follow a similar trend by starting at 1 and 0.99, respectively on day of outcome and reaching its lowest score on day 5 with 0.95 and 0.94, respectively (Figure 7C). It's possible that the 100% accuracy for day 7–12 could be due to the less number of samples, but its worth noting that both classes were present and the model consistently predicted them all correctly for day 7–12 with AUC and F1 scores at 1. Supplementary Figures 5–9 show the performance of the other five algorithms after testing with the three cases.

As mentioned above, the models and their performances presented above were trained based on the test data that was imputed using the training set. Models were also trained using the six different algorithms using the test data without any synthetic data (without imputation) by just simply dropping the data points with missing data. From Supplementary Table 2, it can be observed that again the neural network performed well on the test set with an accuracy of 94.61 ± 0.85%, AUC as 0.98 ± 0.01 and F1 Score of 0.95 ± 0.01. Logistic Regression is the next best performing algorithm with results close to neural network. For robust testing, we again used the three cases (Cases 1, 2, and 3) as discussed above to determine the predictive performances. Supplementary Figures 10–17 show the performance of all the models after testing with the three cases.

## Discussion

The COVID-19 pandemic has put an immense pressure on the healthcare systems around the globe due to the rapid rise in the number of infections. In these times, it is extremely crucial to assess risk such that critical resources can be mobilized to treat patients progressing to severe stages. Focused medical treatments can be administered only when there is a clear understanding of the risk factors that influence the mortality the most. Due to the recent rise of the disease, new features that affect the progression of the disease are continuously being inquired. Machine learning methods are capable of discerning useful patterns in large dimensional data. This study reports machine learning model that is expected to aid in the decision-making process of identifying patients who are at high risk with high accuracy.

**Figure 8:**
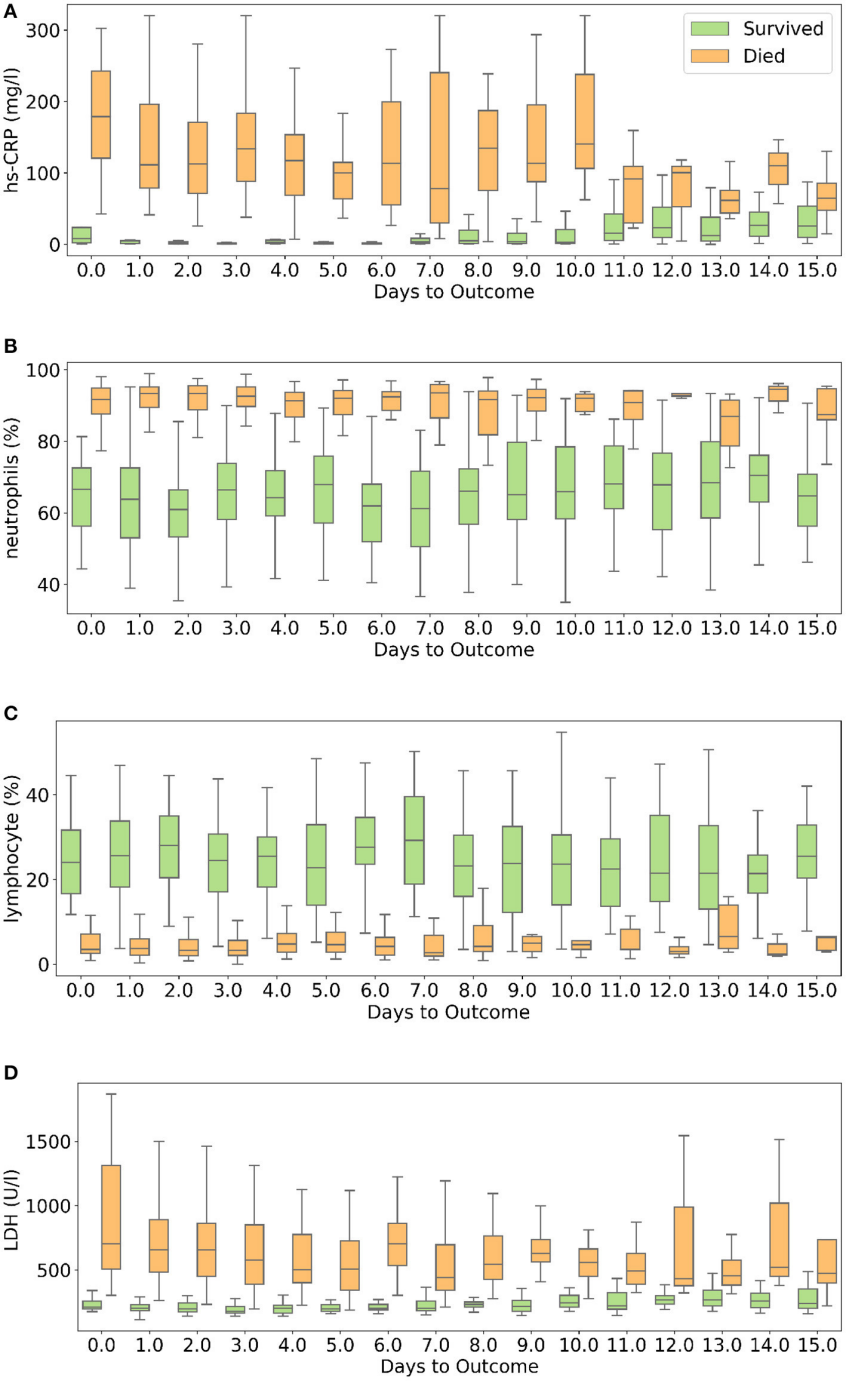
Box and whisker plot showing the variations of four selected features with respect to the days to outcome. **(A)** hs-CRP, **(B)** neutrophils (%), **(C)** lymphocyte (%), **(D)** lactate dehydrogenase.

In this study, XGBoost feature importance and neural network were utilized to find the right balance between high AUC score and low number of features selected for developing the ML models. In this process, five features were chosen to create a powerful combination for mortality prediction. The selected five features include neutrophils (%), hs-CRP, age, lymphocyte (%), and LDH. Each of these features have been identified as predictors of mortality associated with the COVID-19 disease (18, 22, 24, 25, 28–31, 44–46). Age has been identified as an important factor in COVID-19 disease progression and hence it has been included in all the models here (4–6, 22, 24, 25). Patients aged ≥60 years had a higher rate of respiratory failure and needed more prolonged treatment than those aged <60 years (4), implying that the elderly showed poorer response to treatments than the younger age group. Older patients (age ≥80 years) had a risk of 41.3% of having severe or critical condition upon contracting COVID-19 while younger patients (age <20 years) had a lower risk of only 4.1% (5). Older people are also more susceptible to co-morbidities which has been identified as another independent risk factor for COVID-19 disease prognosis (6).

hs-CRP is produced in the liver that responds to a wide range of health conditions, leading to inflammation. Figure 8A shows that people who died had higher levels of hs-CRP than those who survived. Studies have found that higher hs-CRP levels correlates with lower pulmonary functions (47, 48) and patients with Chronic Obstructive Pulmonary Disease (COPD) have been found to be with higher hs-CRP level compared to normal population (49, 50). hs-CRP has also been identified as an important factor to facilitate triage of COVID-19 patients (51). Very high hs-CRP level show the development of severe bacterial superinfection, which is expected to be a frequent complication in critical patients with COVID-19, and potentially a reason for increased mortality. Identifying patients at higher risk of superinfections or other complications before observing substantial increases in hs-CRP and LDH levels may help in treating them more efficiently (52).

Neutrophils are a type of white blood cells and are the first line of defense in the inflammatory response (53). Elevated levels of neutrophils (neutrophilia) suggest that the body is infected. Figure 8B shows that patients who died had significantly higher levels of neutrophils (%) compared to patients who survived. Lymphocyte consist of commonly known B cells, T cells, and NK cells. Figure 8C shows that people who died had significantly lower levels of lymphocyte (%) than those who survived. Neutrophil to Lymphocyte Ratio (NLR) has been identified as an independent risk factor for critical illness in COVID-19 patients (54).

Figure 8D shows that patients who died had higher LDH levels than those who survived. High LDH levels are also linked to co-infection and possibly predict prognosis in severe bacterial infections (52). LDH has been identified as an important indicator of lung damage or inflammation (55). Several studies have identified LDH as an important factor to study COVID-19 disease progression and have linked high levels of LDH to higher risk or severity and fatality (56–58). While it is helpful to provide cutoff values of the features identified here for direct applicability (31), this can vary depending on the type of methods used for measurement. For example, LDH levels cut-off can vary depending on measurement techniques (59). Yan et al. (31) clarified that Tongji Hospital used the conversion of lactate to pyruvate (L → P) with concomitant reduction of NAD^+^ to NADH (60). According to the guidelines of the kit used, the normal range is ≤250 U/l^−1^ in adults. Also, blood samples with haemolysis were not added in the dataset (60).

We compared several machine learning models for their predictive performance with the selected five features. The test set had a good representation of both the survived (43.66%) and dead (56.34%) patients. To realize the actual predictive performance of the models and understand how confident we can be with the results, robust testing was carried out using three cases. It was observed that the neural net consistently performed better than the rest of the models. Neural network was able to predict patients' mortality with an accuracy of 96.53% and an F1-Score of 0.969 on a test set of 213 samples spread across multiple days during the span of the disease. The highly accurate and consistent performance of the neural net based model after robust testing with the five chosen features gives a strong confidence on the model.

Other machine learning models involving trees and regression algorithms performed with an average accuracy of 94%. This shows that the five selected features are extremely influential on patient mortality. The selected features show a clear difference in trend for the two classes though there is a significant overlap between the two sets of data (Figure 8). This explains the decent performance of simple algorithms like Logistic Regression and SVM. Neural Networks move a step ahead by distinguishing the features for the two classes to a greater accuracy and greater consistency through the days. Although the neural networks take time to train, once trained they are able to produce results at speeds comparable to simple algorithms.

We have tested using stratified five-fold cross validation and ensured a good representation of both the classes in the test set (32). Yan et al. (31) only tested their model using Case 1 whereas our model was tested using two more practical cases, and performed more accurately and consistently on Case 1 as well. Our method however has few limitations. The proposed models have been developed based on the data from patients exclusively from a single hospital from Wuhan forcing it to have certain biases including patient management and viral strain found in Wuhan. Mutations might have changed the disease progression patterns in other populations (61–65). Nevertheless, studies done on other cohorts have also identified these features as key predictors (44–46). The dataset unfortunately did not include information on comorbidities. Owing to its importance as a determinant of COVID-19 outcome, we have collected clinical data on Indian COVID19 patients including the details of comorbidities and have developed ML models for both risk stratification and mortality (66–69). After experimenting with various algorithms, we observed that there is a trade-off between accuracy and interpretability. The best performing model, neural network, works like a black-box. However, XGBoost importance graph adds interpretability to it. If the model could be improved using data from diverse sources, implementing either of the types of models in the clinical setting is possible. The currently proposed models are accurate enough to capture the mortality rates and hence can help in targeted caring of high risk patients.

Features other than the five used for developing the reported models were further analyzed to identify the ones that show different trends with respect to the outcome during the span of the disease. Supplementary Figure 18 shows the progression of nine other features that seem to show trends with respect to number of days to outcome. Calcium and serum potassium could be used to predict days until death for critical patients, as it shows an increasing trend in value toward the day of outcome. Platelet count can be used to analyze trends of features in patients belonging to both the classes. The dataset used for this work is insufficient to conduct such a study due to its size and density. We believe that with a larger dataset it may be possible to analyze these trends more meaningfully and possibly predict the number of days to outcome. This can potentially scale up the resource planning by many folds and could prove to be of great significance.

## Summary

In summary, this study reports the identification of powerful combination of five features [neutrophils (%), hs-CRP, age, lymphocyte (%), and LDH] that helps in accurately predicting the mortality of COVID-19 patients. Different machine learning models have been developed to compare and predict mortality most accurately. The neural network predicts the mortality with 96% accuracy for all days during the span of the disease and with 90% accuracy for more than 16 days in advance. XGBoost feature importance provides interpretability to the model that may be relevant in the clinical setting. The robustness of the proposed model was thoroughly tested with three different scenarios. The performance metrics obtained instill great confidence on the proposed model. Other possible features that have predictive capability are identified, however will need data from diverse sources to further confirm their relevance and to possibly improve the model.

## Data Availability Statement

Publicly available datasets were analyzed in this study. This data can be found at: https://github.com/HAIRLAB/Pre_Surv_COVID_19/tree/master/data.

## Author Contributions

AK and AG: implementation of the machine learning models. PV and UP: conceptualization and design of the problem. All the authors contributed to the analysis of the data and wrote the manuscript.

## Conflict of Interest

The authors declare that the research was conducted in the absence of any commercial or financial relationships that could be construed as a potential conflict of interest.

## References

[1] Zu ZY Di Jiang M Xu PP Chen W Ni QQ Lu GM. Coronavirus disease 2019 (covid-19): a perspective from china. Radiology. (2020) 2020:200490. 10.1148/radiol.2020200490 32083985 PMC7233368

[2] Menni C Valdes AM Freidin MB Sudre CH Nguyen LH Frew DA. Real-time tracking of self-reported symptoms to predict potential COVID-19. Nat Med. (2020) 26:1037–40. 10.1038/s41591-020-0916-2 32393804 PMC7751267

[3] Callejon-Leblic MA Moreno-Luna R Del Cuvillo A Reyes-Tejero IM Garcia-Villaran MA Santos-Peña M. Loss of smell and taste can accurately predict COVID-19 infection: a machine-learning approach. J Clin Med. (2021). 10:570. 10.3390/jcm10040570 33546319 PMC7913595

[4] Liu Y Mao B Liang S Yang JW Lu HW Chai YH. Association between age and clinical characteristics and outcomes of COVID-19. Eur Respir J. (2020) 55:2001112. 10.1183/13993003.01112-2020 32312864 PMC7173682

[5] Pan A Liu L Wang C Guo H Hao X Wang Q. Association of public health interventions with the epidemiology of the COVID-19 outbreak in Wuhan, China. JAMA. (2020) 323:1915–23. 10.1001/jama.2020.6130 32275295 PMC7149375

[6] Bajgain KT Badal S Bajgain BB Santana MJ. Prevalence of comorbidities among individuals with COVID-19: a rapid review of current Literature. Am J Infect Control. (2020) 49:238–46. 10.1016/j.ajic.2020.06.213 32659414 PMC7351042

[7] Arpaci I Huang S Al-Emran M Al-Kabi MN Peng M. Predicting the COVID-19 infection with fourteen clinical features using machine learning classification algorithms. Multim Tools Appl. (2021) 80:11943–57. 10.1007/s11042-020-10340-7 33437173 PMC7790521

[8] Wan Y Zhou H Zhang X. An interpretation architecture for deep learning models with the application of COVID-19 diagnosis. Entropy. (2021) 23:204. 10.3390/e23020204 33562309 PMC7916048

[9] Imran A Posokhova I Qureshi HN Masood U Riaz MS Ali K. AI4COVID-19: AI enabled preliminary diagnosis for COVID-19 from cough samples via an app. Inform Med Unlock. (2020) 20:100378. 10.1016/j.imu.2020.100378 32839734 PMC7318970

[10] He X Yang X Zhang S Zhao J Zhao Y Xing E. Sample-efficient deep learning for COVID-19 diagnosis based on CT Scans. MedRxiv [Preprint]. (2020). 10.1101/2020.04.13.20063941

[11] Jamshidi M Lalbakhsh A Talla J Peroutka Z Hadjilooei F Lalbakhsh P. Artificial intelligence and covid-19: deep learning approaches for diagnosis and treatment. IEEE Access (2020) 8:109581–95. 10.1109/ACCESS.2020.3001973 PMC8043506 34192103

[12] Roy S Menapace W Oei S Luijten B Fini E Saltori C. Deep learning for classification and localization of covid-19 markers in point-of-care lung ultrasound. IEEE Trans Med Imaging. (2020) 39:2676–87. 10.1109/TMI.2020.2994459 32406829

[13] Silverstein J. Most of the World Doesn't Have Access to X-Rays. The Atlantic. (2018). Available online at: https://www.theatlantic.com/health/archive/2016/09/radiology-gap/501803/

[14] Kurjak A Breyer B. The use of ultrasound in developing countries. Ultras Med Biol. (1986) 12:611–21. 10.1016/0301-5629(86)90182-1 3532475

[15] Liang W Liang H Ou L Chen B Chen A Li C. Development and validation of a clinical risk score to predict the occurrence of critical illness in hospitalized patients with COVID-19. JAMA Intern Med. (2020) 180:1–9. 10.1001/jamainternmed.2020.2033 32396163 PMC7218676

[16] Lu J Hu S Fan R Liu Z Yin X Wang Q. ACP risk grade: a simple mortality index for patients with confirmed or suspected severe acute respiratory syndrome coronavirus 2 disease (COVID-19) during the early stage of outbreak in Wuhan, China. MedRxiv [Preprint]. (2020). 10.1101/2020.02.20.20025510

[17] Gemmar P. An interpretable mortality prediction model for COVID-19 patients-alternative approach. MedRxiv [Preprint]. (2020). 10.1101/2020.06.14.20130732

[18] Chowdhury MEH Rahman T Khandakar A Al-Madeed S Zughaier SM Doi SM. An early warning tool for predicting mortality risk of covid-19 patients using machine learning. arXiv preprint arXiv:2007.15559. (2020). 10.1007/s12559-020-09812-7 PMC8058759 33897907

[19] Kang J Chen T Luo H Li L Yang MJ. Machine learning predictive model for severe covid-19. Infect Genet Evol. (2021) 90:104737. 10.1016/j.meegid.2021.104737 33515712 PMC7840410

[20] Pourhomayoun M Shakibi M. Predicting mortality risk in patients with COVID-19 using machine learning to help medical decision-making. Smart Health. (2021) 20:100178. 10.1016/j.smhl.2020.100178 33521226 PMC7832156

[21] Schöning V Liakoni E Baumgartner C Exadaktylos AK Hautz WE Atkinson A. Development and validation of a prognostic COVID-19 severity assessment (COSA) score and machine learning models for patient triage at a tertiary hospital. J Transl Med. (2021) 19:56. 10.1186/s12967-021-02720-w 33546711 PMC7862984

[22] Bolourani S Brenner M Wang P McGinn T Hirsch JS Barnaby D. A machine learning prediction model of respiratory failure within 48 hours of patient admission for COVID-19: model development and validation. J Med Intern Res. (2021) 23:e24246. 10.2196/24246 33476281 PMC7879728

[23] Xu W Sun NN Gao HN Chen ZY Yang Y Ju B. Risk factors analysis of COVID-19 patients with ards and prediction based on machine learning. Sci Rep. (2021) 11:2933. 10.1038/s41598-021-82492-x 33536460 PMC7858607

[24] Jimenez-Solem E Petersen TS Hansen C Hansen C Lioma C Igel C. Developing and validating COVID-19 adverse outcome risk prediction models from a bi-national european cohort of 5594 patients. Sci Rep. (2021) 11:3246. 10.1038/s41598-021-81844-x 33547335 PMC7864944

[25] Ikemura K Bellin E Yagi Y Billett H Saada M Simone K. Using automated-machine learning to predict COVID-19 patient mortality. J Med Intern Res. (2021) 23:e23458. 10.2196/23458 PMC7919846 33539308

[26] Ji D Zhang D Xu J Chen Z Yang T Zhao P. Prediction for progression risk in patients with COVID-19 pneumonia: the CALL Score. Clin Infect Dis. (2020) 71:1393–9. 10.1093/cid/ciaa414 32271369 PMC7184473

[27] Hao B Sotudian S Wang T Xu T Hu T Gaitanidis A. Early prediction of level-of-care requirements in patients with COVID-19. Elife. (2020) 9:e60519. 10.7554/eLife.60519 33044170 PMC7595731

[28] Wang K Zuo P Liu Y Zhang M Zhao X Xie S. Clinical and laboratory predictors of in-hospital mortality in patients with COVID-19: a cohort study in Wuhan, China. Clin Infect Dis. (2020) 71:2079–88. 10.1093/cid/ciaa538 32361723 PMC7197616

[29] Shang Y Liu T Wei Y Li J Shao L Liu M. Scoring systems for predicting mortality for severe patients with COVID-19. EClinicalMedicine. (2020) 24:100426. 10.1016/j.eclinm.2020.100426 32766541 PMC7332889

[30] Xie J Hungerford D Chen H Abrams ST Li S Wang G. Development and external validation of a prognostic multivariable model on admission for hospitalized patients with COVID-19. medRxiv [Preprint]. (2020). 10.1101/2020.03.28.20045997

[31] Yan L Zhang HT Yuan Y. An interpretable mortality prediction model for COVID-19 patients. Nat Mach Intell. (2020) 2:283–8. 10.1038/s42256-020-0180-7

[32] Huang C Long X Zhan Z van den Heuvel E. Model stability of COVID-19 mortality prediction with biomarkers. medRxiv [Preprint]. (2020). 10.1101/2020.07.29.20161323

[33] Chen T Guestrin C. Xgboost: a scalable tree boosting system. In: Proceedings of the 22nd ACM SIGKDD International Conference on Knowledge Discovery and Data Mining (2016). p. 785–94. 32561836

[34] Elith J Leathwick JR Hastie T. A working guide to boosted regression trees. J Anim Ecol. (2008) 77:802–13. 10.1111/j.1365-2656.2008.01390.x 18397250

[35] Dreiseitl S Ohno-Machado L. Logistic regression and artificial neural network classification models: a methodology review. J Biomed Inform. (2002) 35:352–9. 10.1016/S1532-0464(03)00034-0 12968784

[36] Wright RE. Logistic regression. In: Grimm LG Yarnold PR, editors. Reading and Understanding Multivariate Statistics. American Psychological Association (1995). p. 217–44.

[37] Hosmer DW Jr Lemeshow S Sturdivant RX. Applied Logistic Regression. Vol. 398 (Hoboken, NJ: John Wiley & Sons 2013).

[38] Rehman A Khan MA Mehmood Z Saba T Sardaraz M Rashid M. Microscopic melanoma detection and classification: a framework of pixel-based fusion and multilevel features reduction. Microsc Res Techn. (2020) 83:410–23. 10.1002/jemt.23429 31898863

[39] Breiman L. Random forests. Mach Learn. (2001) 45:5–32. 10.1023/A:1010933404324

[40] Khan SA Nazir M Khan MA Saba T Javed K Rehman A. Lungs nodule detection framework from computed tomography images using support vector machine. Microsc Res Techn. (2019) 82:1256–66. 10.1002/jemt.23275 30974031

[41] Saba T. Computer vision for microscopic skin cancer diagnosis using handcrafted and non-handcrafted features. Microsc Res Techn. (2021). 10.1002/jemt.23686 33399251

[42] Khan MA Sharif M Akram T Raza M Saba T Rehman A. Hand-crafted and deep convolutional neural network features fusion and selection strategy: an application to intelligent human action recognition. Appl Soft Comput. (2020) 87:105986. 10.1016/j.asoc.2019.105986

[43] Hansen LK Salamon P. Neural network ensembles. IEEE Trans Pattern Anal Mach Intell. (1990) 12:993–1001.

[44] Vaid A Somani S Russak AJ De Freitas JK Chaudhry FF Paranjpe I. Machine learning to predict mortality and critical events in a cohort of patients with COVID-19 in New York city: model development and validation. J Med Internet Res. (2020) 22:e24018. 10.2196/24018 33027032 PMC7652593

[45] De Smet R Mellaerts B Vandewinckele H Lybeert P Frans E Ombelet S. Frailty and mortality in hospitalized older adults with COVID-19: retrospective observational study. J Am Med Direct Assoc. (2020) 21:928–32.e1. 10.1016/j.jamda.2020.06.008 32674821 PMC7280137

[46] Bhandari S Shaktawat AS Tak A Patel B Shukla J Singhal S. Logistic regression analysis to predict mortality risk in covid-19 patients from routine hematologic parameters. Ibnosina J Med Biomed Sci. (2020) 12:123. 10.4103/ijmbs.ijmbs_58_20

[47] Aronson D Roterman I Yigla M Kerner A Avizohar O Sella R. Inverse association between pulmonary function and C-reactive protein in apparently healthy subjects. Am J Respir Crit Care Med. (2006) 174:626–32. 10.1164/rccm.200602-243OC 16778162

[48] Rasmussen F Mikkelsen D Hancox RJ Lambrechtsen J Nybo M Hansen HS. High-sensitive C-reactive protein is associated with reduced lung function in young adults. Eur Respir J. (2009) 33:382–8. 10.1183/09031936.00040708 19010993

[49] Agarwal R Zaheer MS Ahmad Z Akhtar J. The relationship between C-reactive protein and prognostic factors in chronic obstructive pulmonary disease. Multidiscip Respir Med. (2013) 8:63. 10.1186/2049-6958-8-63 24073993 PMC3844686

[50] Nillawar AN Joshi KB Patil SB Bardapurkar JS Bardapurkar SJ. Evaluation of HS-CRP and lipid profile in COPD. J Clin Diagn Res. (2013) 7:801–3. 10.7860/JCDR/2013/5187.2943 23814714 PMC3681041

[51] Li Q Ding X Xia G Chen HG Chen F Geng Z. Eosinopenia and elevated C-reactive protein facilitate triage of COVID-19 patients in fever clinic: a retrospective case-control study. EClinicalMedicine. (2020) 23:100375. 10.1016/j.eclinm.2020.100375 32368728 PMC7196382

[52] Giacobbe DR. Clinical interpretation of an interpretable prognostic model for patients with COVID-19. Nat Mach Intell. (2020) 3:16. 10.1038/s42256-020-0207-0

[53] Uhl B Vadlau Y Zuchtriegel G Nekolla K Sharaf K Gaertner F. Aged neutrophils contribute to the first line of defense in the acute inflammatory response. Blood. (2016) 128:2327–37. 10.1182/blood-2016-05-718999 27609642 PMC5122310

[54] Liu J Liu Y Xiang P Pu L Xiong H Li C. Neutrophil-to-lymphocyte ratio predicts critical illness patients with 2019 coronavirus disease in the early stage. J Transl Med. (2020) 18:1–12. 10.1186/s12967-020-02374-0 32434518 PMC7237880

[55] Drent M Cobben N Henderson R Wouters E van Dieijen-Visser M. Usefulness of lactate dehydrogenase and its isoenzymes as indicators of lung damage or inflammation. Eur Respir J. (1996) 9:1736–1742. 8866602 10.1183/09031936.96.09081736

[56] Shi J Li Y Zhou X Zhang Q Ye X Wu Z. Lactate dehydrogenase and susceptibility to deterioration of mild COVID-19 patients: a multicenter nested case-control study. BMC Med. (2020) 18:168. 10.1186/s12916-020-01633-7 32493370 PMC7268591

[57] Wu MY Yao L Wang Y Zhu XY Wang XF Tang PJ. Clinical evaluation of potential usefulness of serum lactate dehydrogenase (LDH) in 2019 novel coronavirus (COVID-19) pneumonia. Respir Res. (2020) 21:171. 10.1186/s12931-020-01427-8 32631317 PMC7336103

[58] Henry BM Aggarwal G Wong J Benoit S Vikse J Plebani M. Lactate dehydrogenase levels predict coronavirus disease 2019 (COVID-19) severity and mortality: a pooled analysis. Am J Emerg Med. (2020) 38:1722–6. 10.1016/j.ajem.2020.05.073 32738466 PMC7251362

[59] Reeve JLV Twomey PJ. Consider laboratory aspects in developing patient prediction models. Nat Mach Intell. (2020) 3:2522–5839. 10.1038/s42256-020-0221-2

[60] Yan L Goncalves J Zhang HT Li S Yuan Y. Reply to: Consider the laboratory aspects in developing patient prediction models. Nat Mach Intell. (2021). 3:19. 10.1038/s42256-020-0220-3

[61] Becerra-Flores M Cardozo T. SARS-CoV-2 viral spike G614 mutation exhibits higher case fatality rate. Int J Clin Pract. (2020) 74:e13525. 10.1111/ijcp.13525 32374903 PMC7267315

[62] Saha P Banerjee AK Tripathi PP Srivastava AK Ray U. A virus that has gone viral: amino acid mutation in S protein of Indian isolate of Coronavirus COVID-19 might impact receptor binding, and thus, infectivity. Biosci Rep. (2020) 40:BSR20201312. 10.1042/BSR20201312 32378705 PMC7225408

[63] Wang R Hozumi Y Yin C Wei GW. Mutations on COVID-19 diagnostic targets. Genomics. (2020) 112:5204–13. 10.1016/j.ygeno.2020.09.028 32966857 PMC7502284

[64] Korber B Fischer WM Gnanakaran S Yoon H Theiler J Abfalterer W. Tracking changes in SARS-CoV-2 Spike: evidence that D614G increases infectivity of the COVID-19 virus. Cell. [Preprint] (2020) 482:812–27. 10.1016/j.cell.2020.06.043 32697968 PMC7332439

[65] Lee HY Perelson AS Park SC Leitner T. Dynamic correlation between intrahost HIV-1 quasispecies evolution and disease progression. PLoS Comput Biol. (2008) 4:e1000240. 10.1371/journal.pcbi.1000240 19079613 PMC2602878

[66] Wang B Li R Lu Z Huang Y. Does comorbidity increase the risk of patients with COVID-19: evidence from meta-analysis. Aging. (2020) 12:6049–57. 10.18632/aging.103000 32267833 PMC7185114

[67] Sanyaolu A Okorie C Marinkovic A Patidar R Younis K Desai P.. Comorbidity and its impact on patients with covid-19. SN Comprehens Clin Med. (2020) 2:1–8. 10.1007/s42399-020-00363-4 32838147 PMC7314621

[68] Guan WJ Liang W Zhao Y Liang H Chen Z Li Y.. Comorbidity and its impact on 1590 patients with COVID-19 in China: a nationwide analysis. Eur Respir J. (2020) 5:2000547. 10.1183/13993003.00547-2020 32217650 PMC7098485

[69] Alle S Siddiqui S Kanakan A Garg A Karthikeyan A Mishra N. COVID-19 risk stratification and mortality prediction in hospitalized Indian patients. medRxiv [Preprint]. (2020). 10.1101/2020.12.19.20248524 PMC8929610 35298502

