# Supplementary material for: Machine Learning Based Clinical Decision Support System for Early COVID-19 Mortality Prediction
Source: Front Public Health. 2021 May 12;9:626697. doi: 10.3389/fpubh.2021.626697 (PMC8149622; doi:10.3389/fpubh.2021.626697)
Supplement: Supplementary file 1 [file Data_Sheet_1.PDF]

.

Supporting Information

Machine learning based clinical decision support system for early COVID-19 mortality prediction

Akshaya Karthikeyan[+], Akshit Garg[+], P. K. Vinod[*], and U. Deva Priyakumar[*]

Center for Computational Natural Sciences and Bioinformatics, International Institute of Information Technology, Hyderabad 500 032, India

[+]Authors contributed equally

TABLES

Model	Mean Accuracy% (std)	Mean F1 Score (std)	Mean AUC (std)
Neural Net	96.526 (0.637)	0.9687 (0.006)	0.9895 (0.0057)
SVM	95.305 (0.514)	0.9577 (0.0046)	0.9903 (0.0014)
Logistic Regression	94.929 (0.188)	0.9537 (0.0018)	0.9934 (0.00015)
Random Forests	94.178 (0.703)	0.9467 (0.0065)	0.9858 (0.0020)
XGBoost	93.709 (1.3477)	0.9435 (0.0118)	0.9838 (0.0022)
Decision Tree	90.892 (1.7963)	0.914 (0.0182)	0.9771 (0.0033)

Table S1. Performance of various algorithms on the imputed test set

Model	Mean Accuracy% (std)	Mean F1 Score (std)	Mean AUC (std)
Neural Net	94.608 (0.852)	0.946 (0.009)	0.982 (0.0086)
Logistic Regression	93.043 («0.01)	0.9298 («0.01)	0.987 (0.00044)
SVM	92.348 (0.852)	0.9239 (0.0086)	0.980 (0.0032)
Random Forests	91.826 (1.301)	0.917 (0.013)	0.972 (0.004)
XGBoost	90.608 (2.0132)	0.9085 (0.0182)	0.969 (0.0026)
Decision Tree	89.0435 (2.101)	0.8863 (0.0223)	0.9615 (0.0057)

Table S2. Performance of various algorithms on test set without imputation

FIGURES

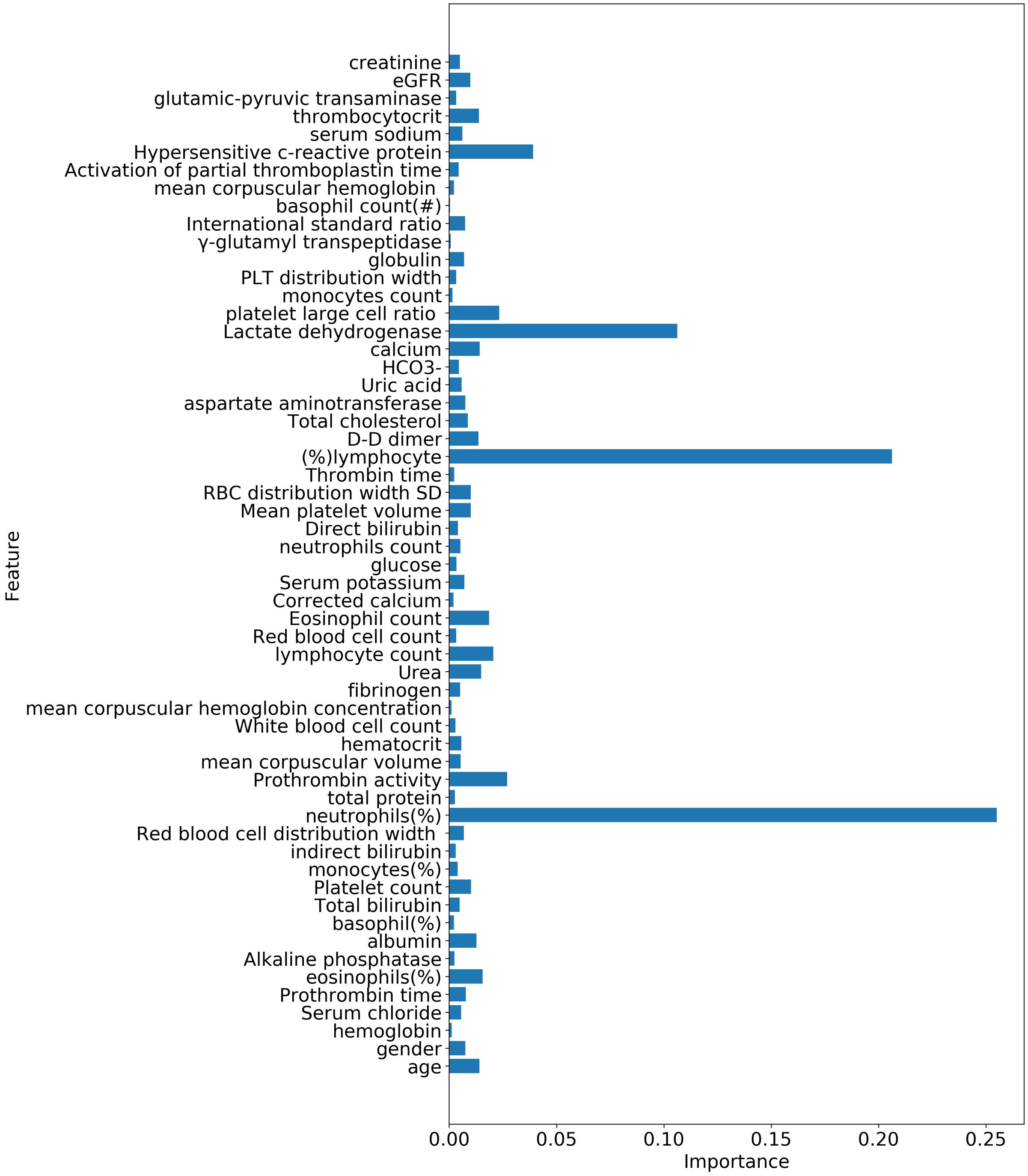

Figure S1. Mean relative importance of all the features in the train set determined using XGBoost. It is shown that the top four important features are neutrophils (%), lymphocyte (%), LDH and hs-CRP

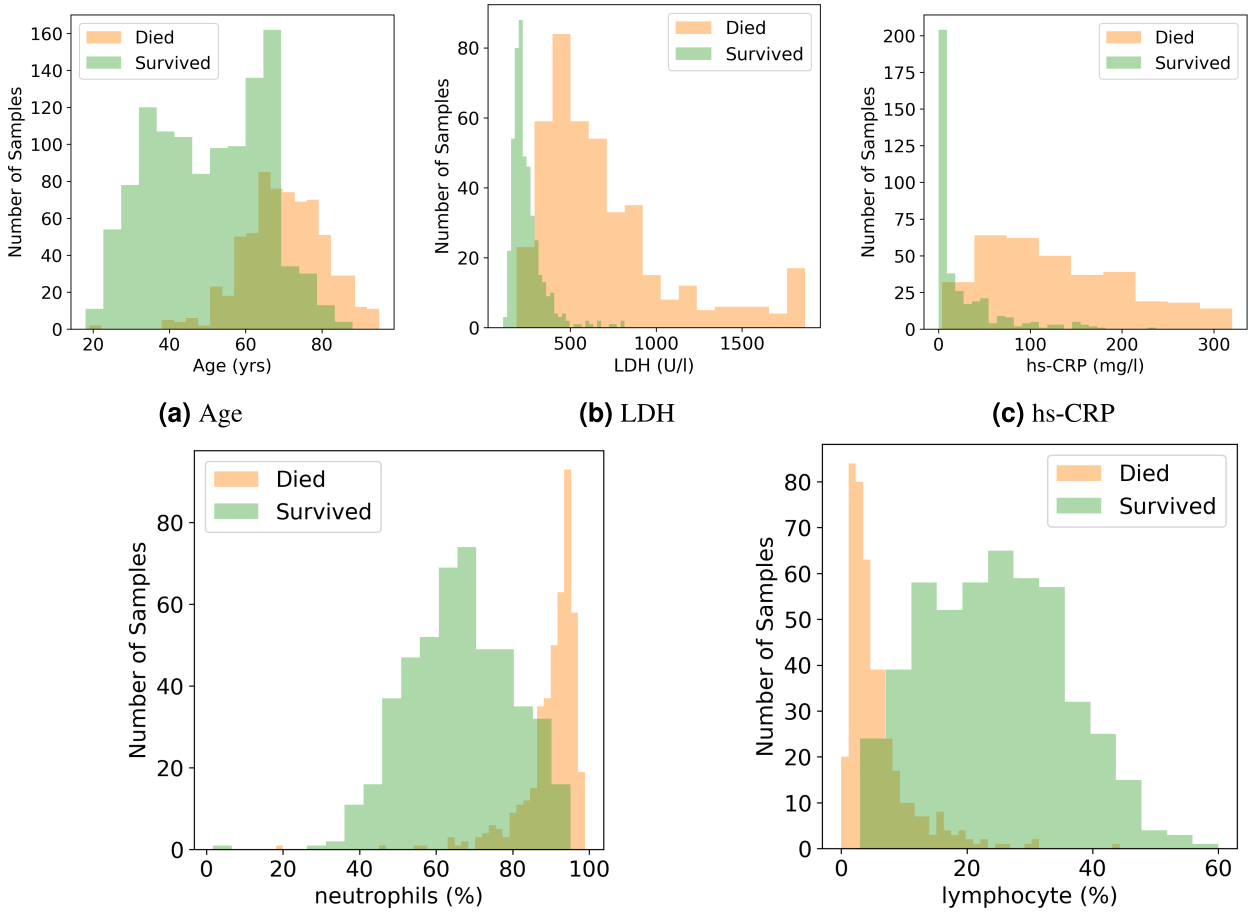

(a) Age **(b)** LDH **(c)** hs-CRP

(d) Neutrophils (%) **(e)** Lymphocytes (%)

Figure S2. Distribution of the five selected features with respect to both the classes- survived and dead.

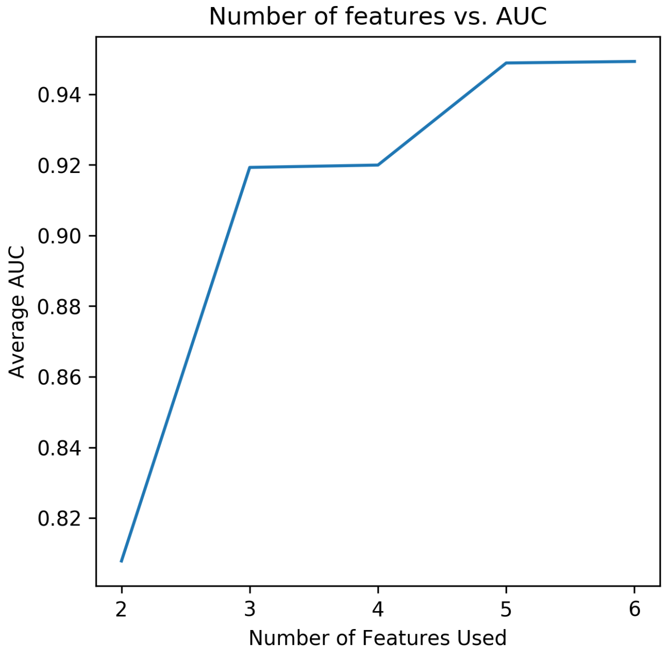

Figure S3. Number of features chosen vs. average AUC score obtained using neural network for feature selection. This suggests using the set of first five features after ordering the features in descending order of their relative importance. Selected five features are: age, neutrophils (%), lymphocytes (%), LDH and hs-CRP

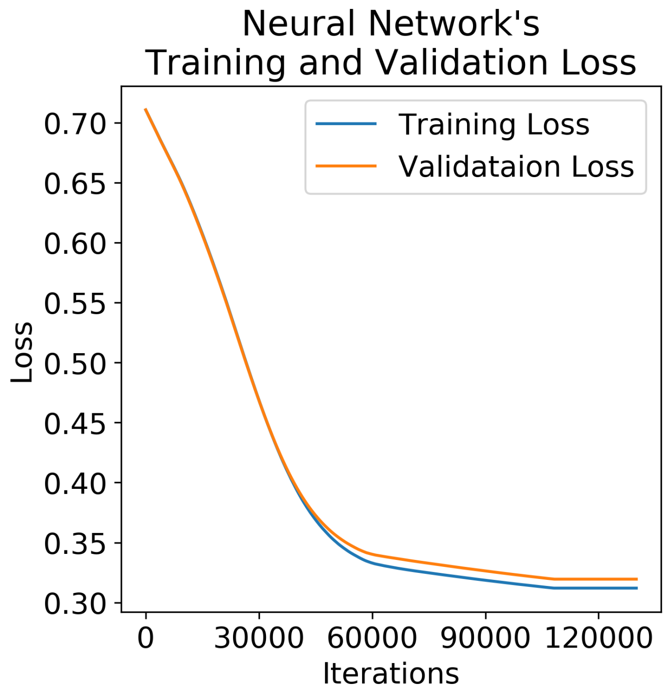

Figure S4. Training and Validation Loss curves for the Neural Network. Here, the number of iterations represent epochs since the data was not divided into batches

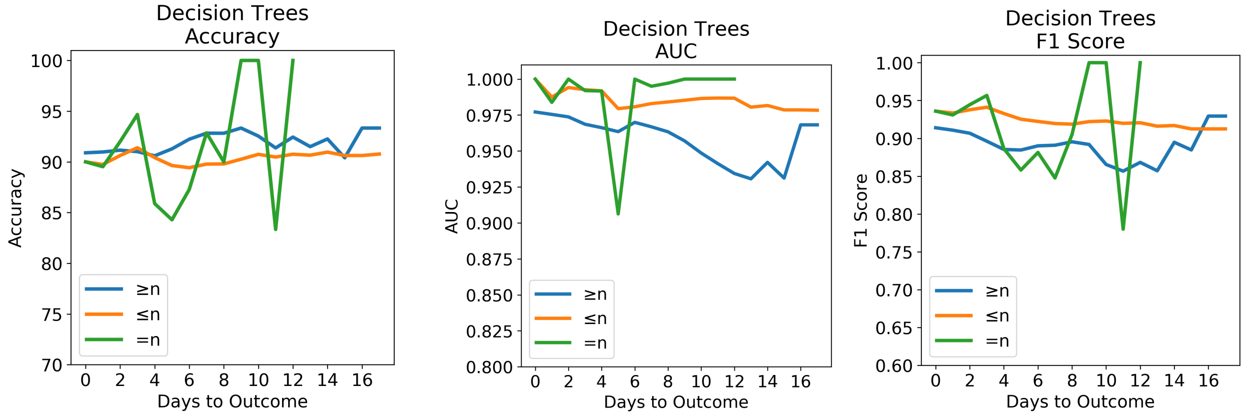

(a) Accuracy **(b)** AUC **(c)** F1-Score

Figure S5. The performance of Decision Trees on the imputed test set using the three testing cases. (Cases ≥n, ≤n and =n) (a) Accuracy of model evaluated for different days to outcome. (b) AUC score of model evaluated for different days to outcome. (c) F1 score of model evaluated for different days to outcome.

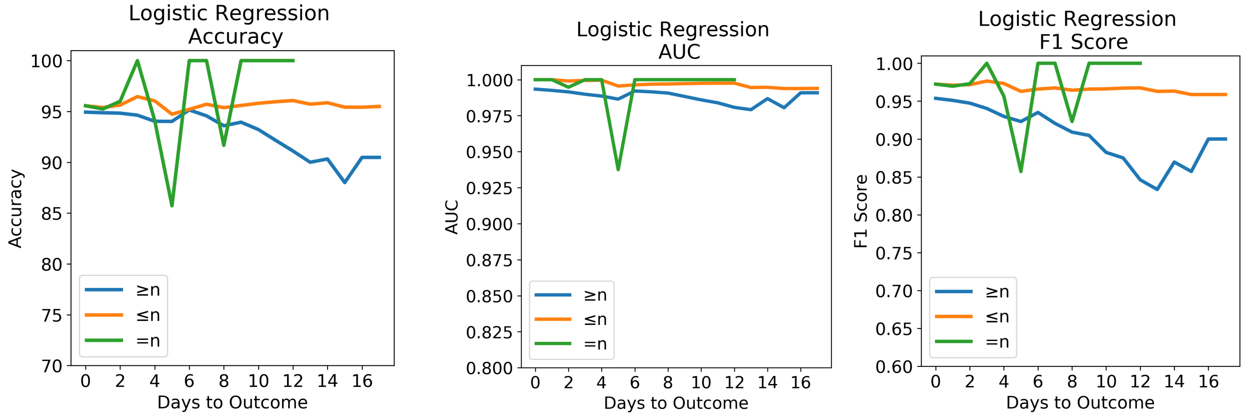

(a) Accuracy **(b)** AUC **(c)** F1-Score

Figure S6. The performance of Logistic Regression on the imputed test set using the three testing cases. (Cases ≥n, ≤n and =n) (a) Accuracy of model evaluated for different days to outcome. (b) AUC score of model evaluated for different days to outcome. (c) F1 score of model evaluated for different days to outcome.

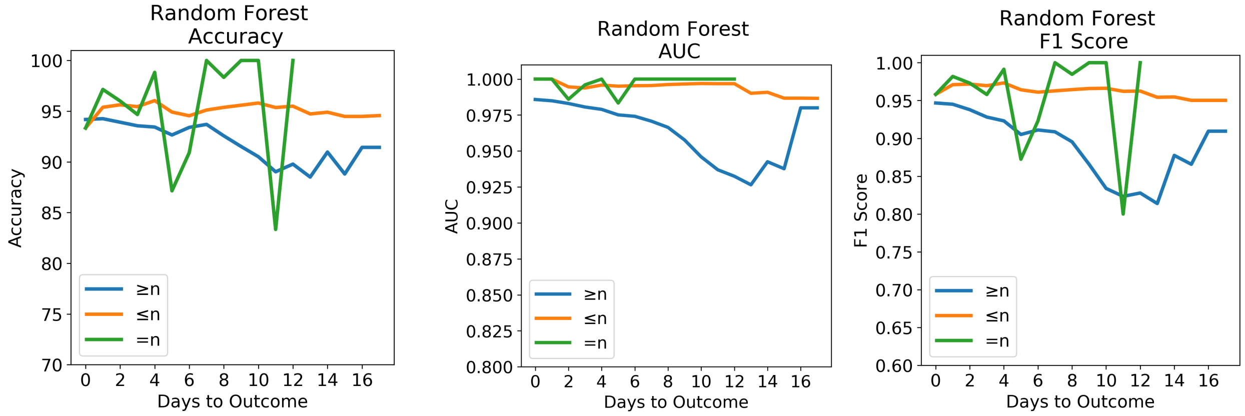

(a) Accuracy **(b)** AUC **(c)** F1-Score

Figure S7. The performance of Random Forests on the imputed test set using the three testing cases. (Cases ≥n, ≤n and =n) (a) Accuracy of model evaluated for different days to outcome. (b) AUC score of model evaluated for different days to outcome. (c) F1 score of model evaluated for different days to outcome.

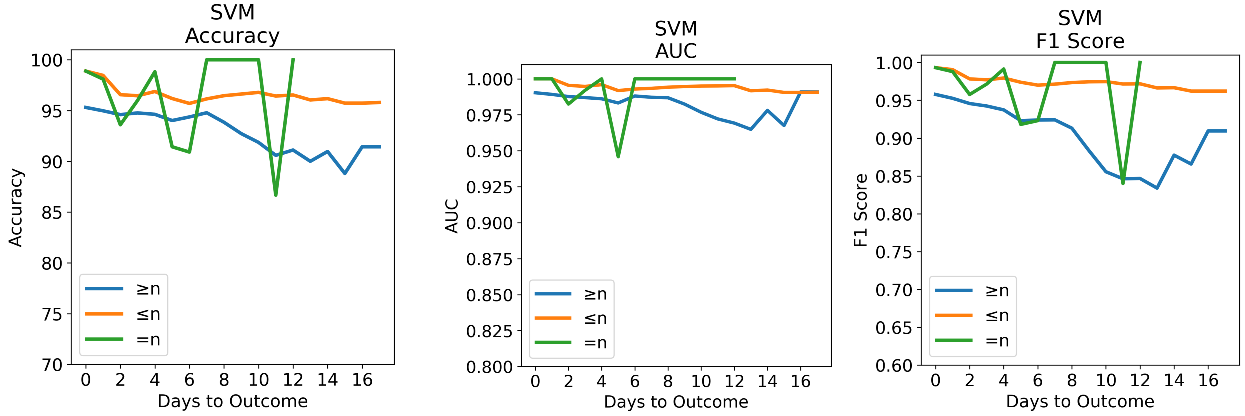

(a) Accuracy **(b)** AUC **(c)** F1-Score

Figure S8. The performance of SVM on the imputed test set using the three testing cases. (Cases ≥n, ≤n and =n) (a) Accuracy of model evaluated for different days to outcome. (b) AUC score of model evaluated for different days to outcome. (c) F1 score of model evaluated for different days to outcome.

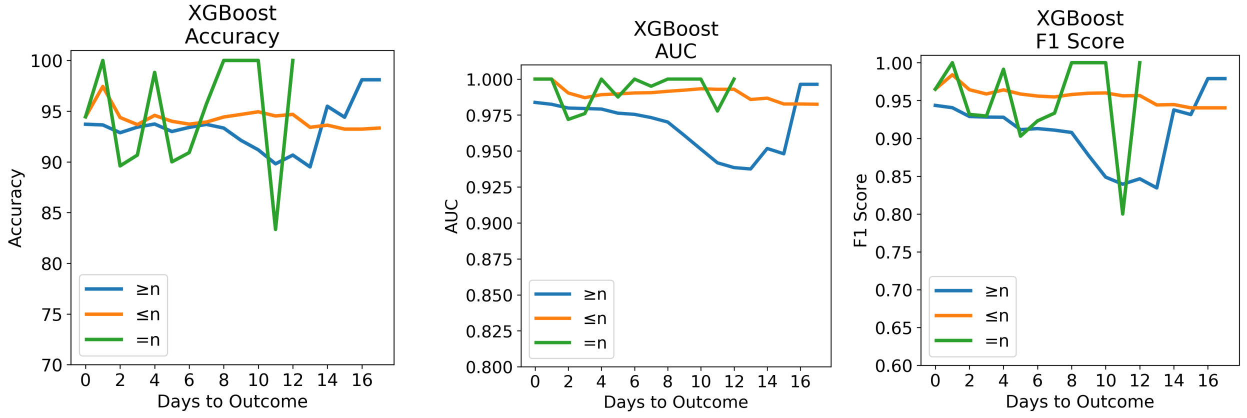

(a) Accuracy **(b)** AUC **(c)** F1-Score

Figure S9. The performance of XGBoost on the imputed test set using the three testing cases. (Cases ≥n, ≤n and =n) (a) Accuracy of model evaluated for different days to outcome. (b) AUC score of model evaluated for different days to outcome. (c) F1 score of model evaluated for different days to outcome.

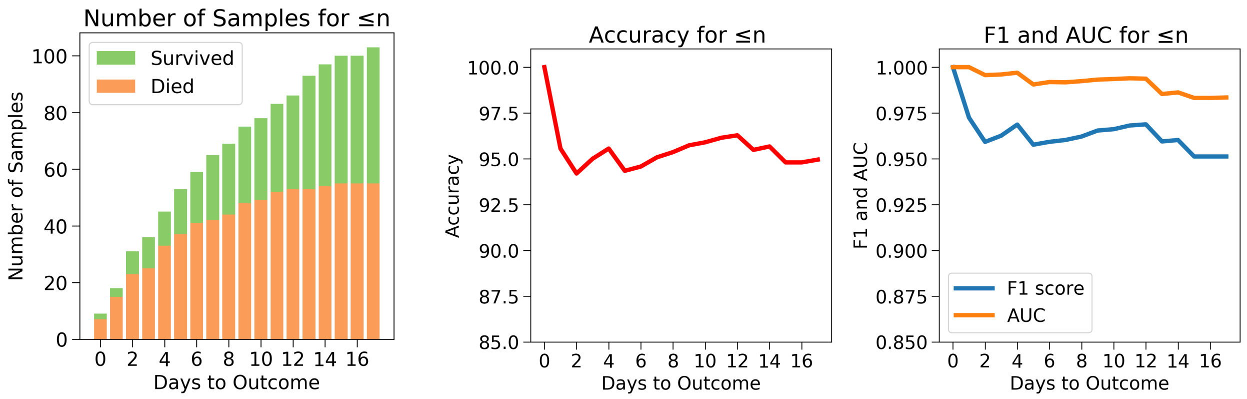

(a) Sample Distribution **(b)** Accuracy **(c)** F1-Score and AUC

Figure S10. The performance of neural net on the test data without imputation using case 1: Number of days to outcome less than or equal to n. (a) The class-wise distribution of the cumulated data-points ($\leq n^{th} day$) for all samples in the test set without imputation. (b) Accuracy of the model evaluated for different days to outcome. (c) F1-score and AUC of the model evaluated for different days to outcome.

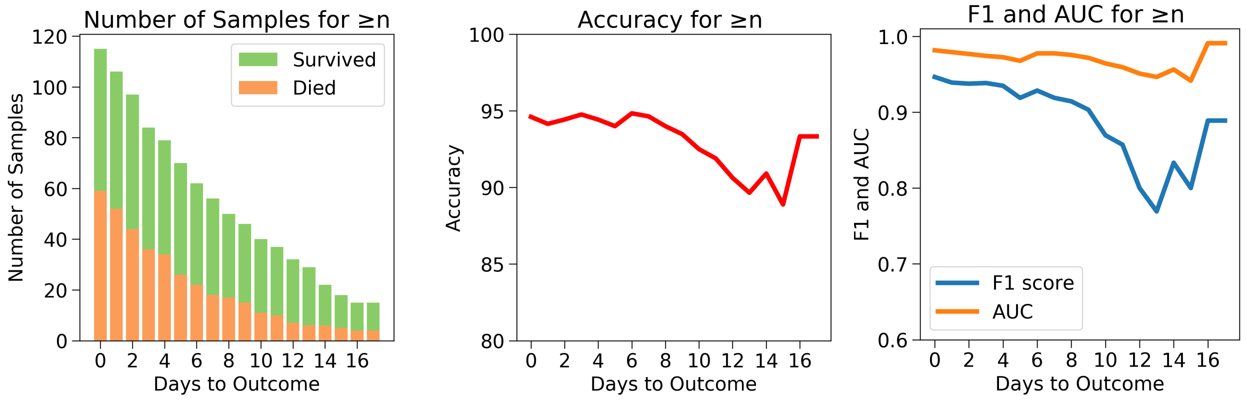

(a) Sample Distribution **(b)** Accuracy **(c)** F1-Score and AUC

Figure S11. The performance of neural net on the test data without imputation using case 2: Number of days to outcome greater than or equal to n. (a) The class-wise distribution of the cumulated data-points ($\leq n^{th} day$) for all samples in the test set without imputation. (b) Accuracy of the model evaluated for different days to outcome. (c) F1-score and AUC of the model evaluated for different days to outcome.

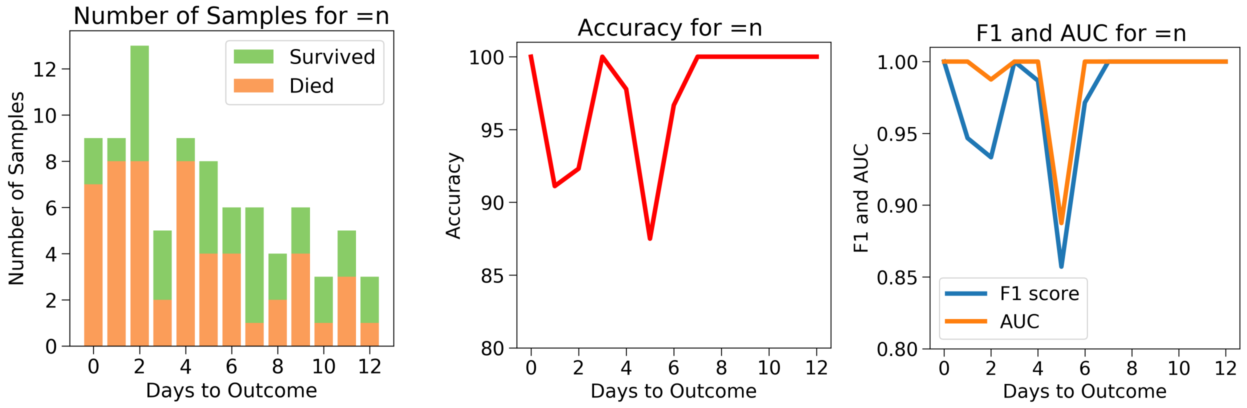

(a) Sample Distribution **(b)** Accuracy **(c)** F1-Score and AUC

Figure S12. The performance of neural net on the test data without imputation using case 3: Number of days to outcome equal to n. (a) The class-wise distribution of the cumulated data-points ($\leq n^{th} day$) for all samples in the test set without imputation. (b) Accuracy of the model evaluated for different days to outcome. (c) F1-score and AUC of the model evaluated for different days to outcome.

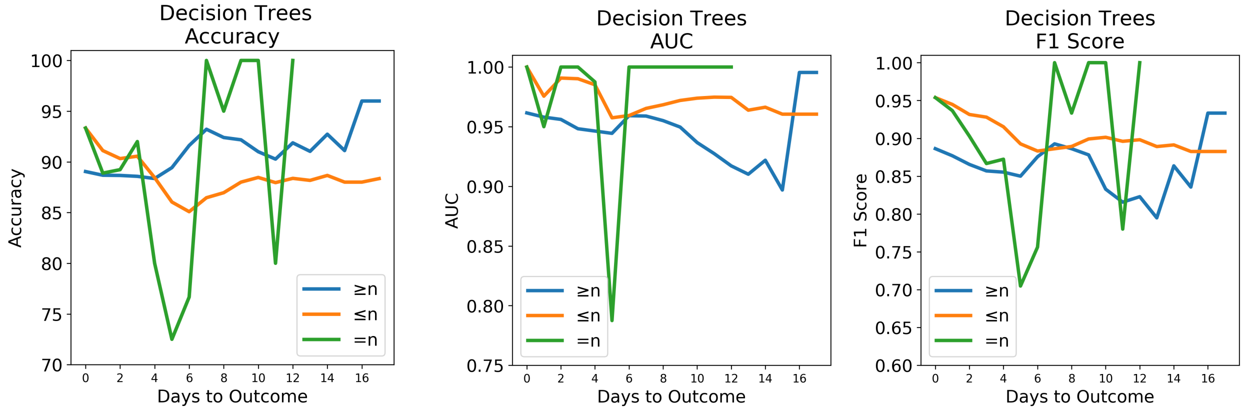

(a) Accuracy **(b)** AUC **(c)** F1-Score

Figure S13. The performance of Decision Trees on the test set without imputation using the three testing cases. (Cases ≥n, ≤n and =n) (a) Accuracy of model evaluated for different days to outcome. (b) AUC score of model evaluated for different days to outcome. (c) F1 score of model evaluated for different days to outcome.

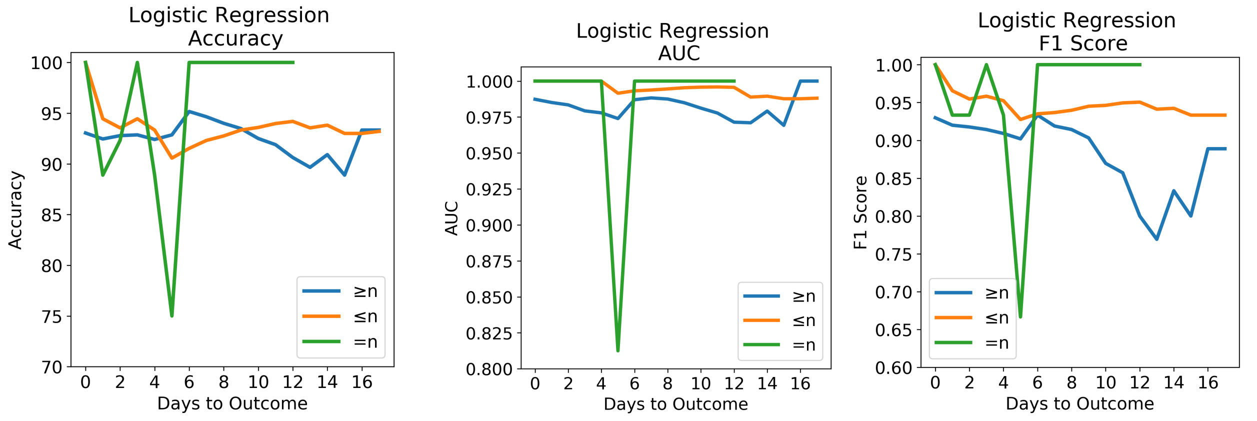

(a) Accuracy **(b)** AUC **(c)** F1-Score

Figure S14. The performance of Logistic Regression on the test set without imputation using the three testing cases. (Cases ≥n, ≤n and =n) (a) Accuracy of model evaluated for different days to outcome. (b) AUC score of model evaluated for different days to outcome. (c) F1 score of model evaluated for different days to outcome.

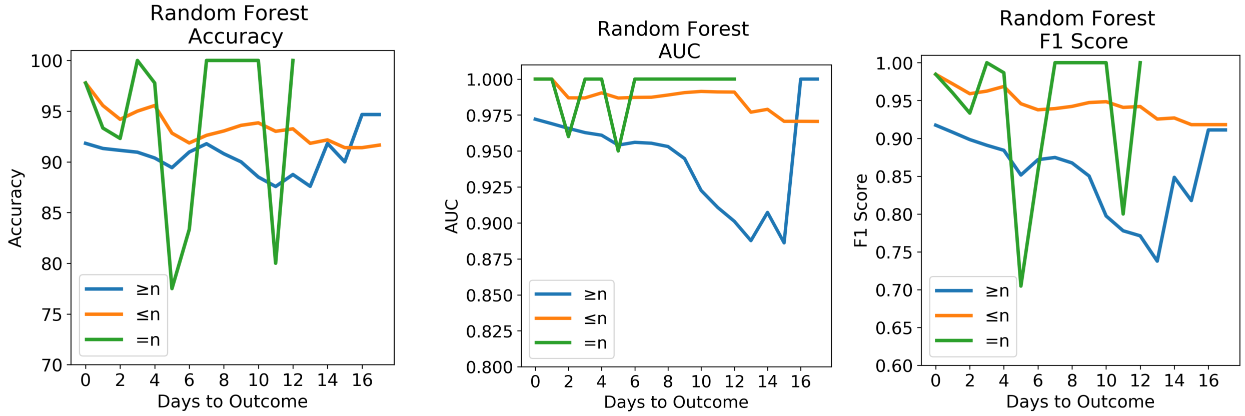

(a) Accuracy **(b)** AUC **(c)** F1-Score

Figure S15. The performance of Random Forests on the test set without imputation using the three testing cases. (Cases $\geq$n, $\leq$n and =n) (a) Accuracy of model evaluated for different days to outcome. (b) AUC score of model evaluated for different days to outcome. (c) F1 score of model evaluated for different days to outcome.

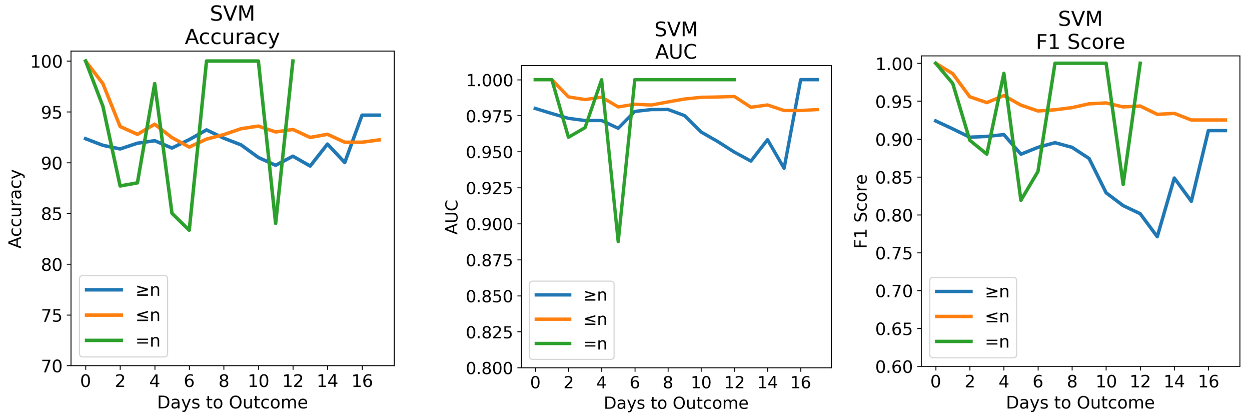

(a) Accuracy **(b)** AUC **(c)** F1-Score

Figure S16. The performance of SVM on the test set without imputation using the three testing cases. (Cases $\geq$n, $\leq$n and =n) (a) Accuracy of model evaluated for different days to outcome. (b) AUC score of model evaluated for different days to outcome. (c) F1 score of model evaluated for different days to outcome.

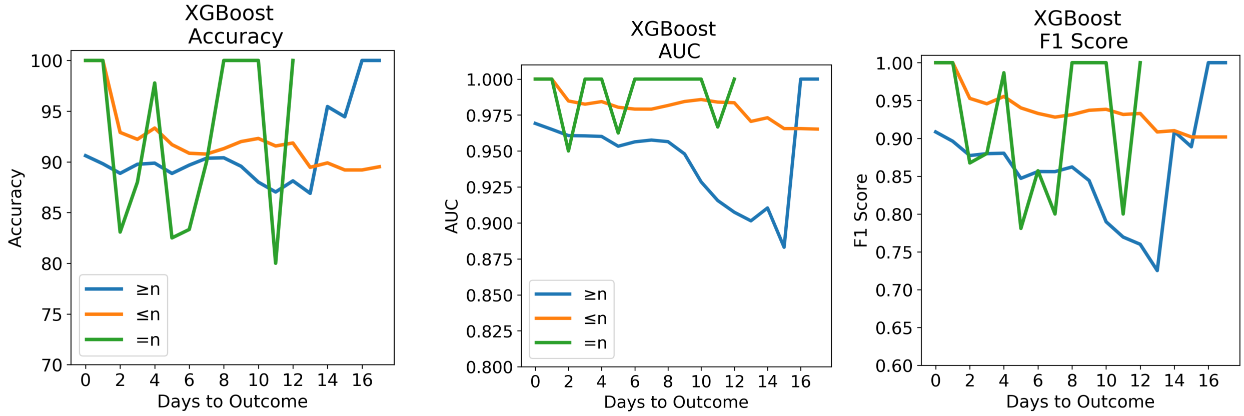

(a) Accuracy **(b)** AUC **(c)** F1-Score

Figure S17. The performance of XGBoost on the test set without imputation using the three testing cases. (Cases $\geq$n, $\leq$n and =n) (a) Accuracy of model evaluated for different days to outcome. (b) AUC score of model evaluated for different days to outcome. (c) F1 score of model evaluated for different days to outcome.

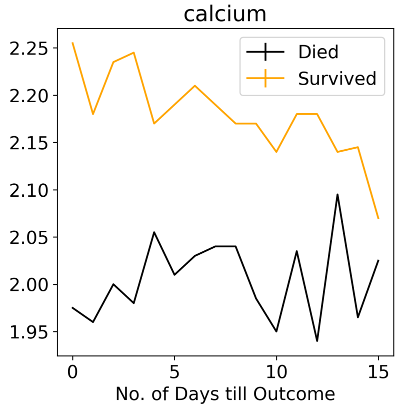

(a) Calcium

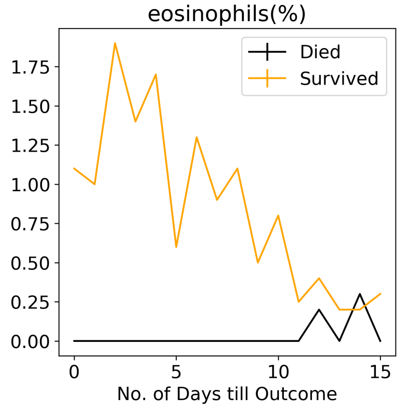

(b) Eosinphils (%)

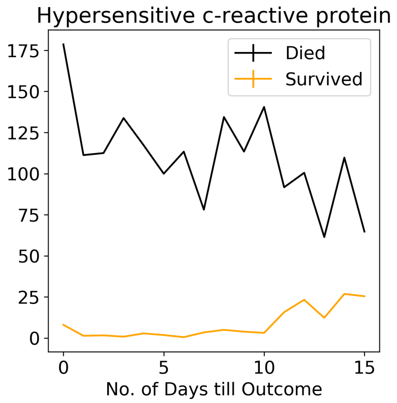

(c) Hs-CRP

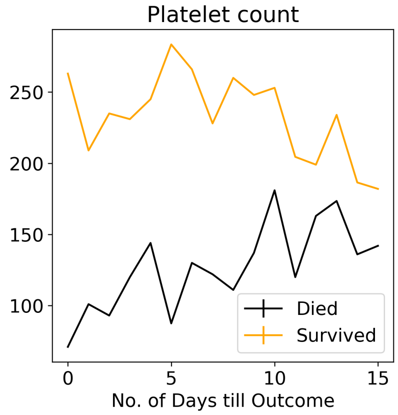

(d) Platelet Count

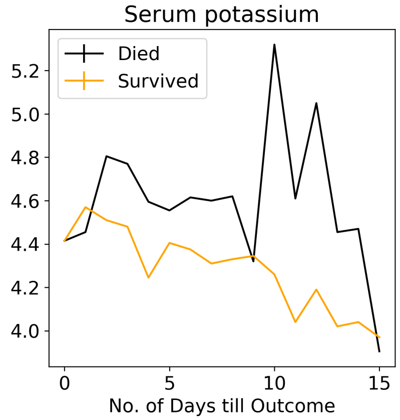

(e) Serum Potassium

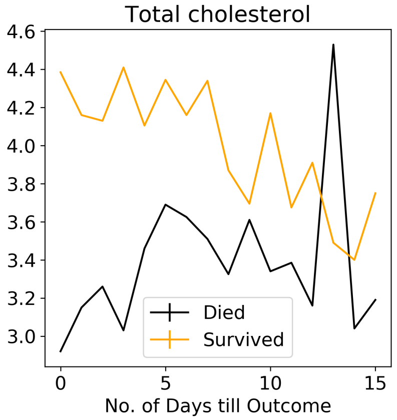

(f) Total Cholesterol

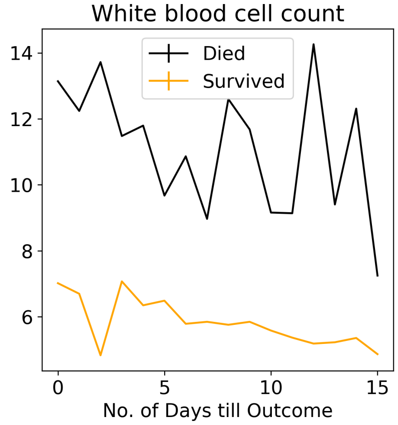

(g) WBC Count

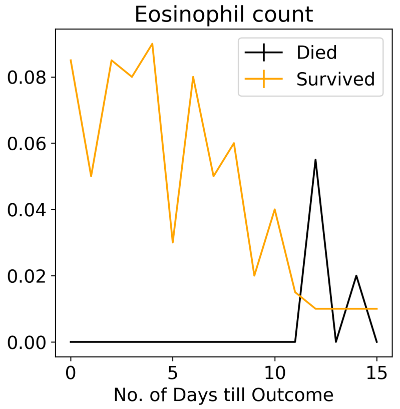

(h) Eosinophil Count

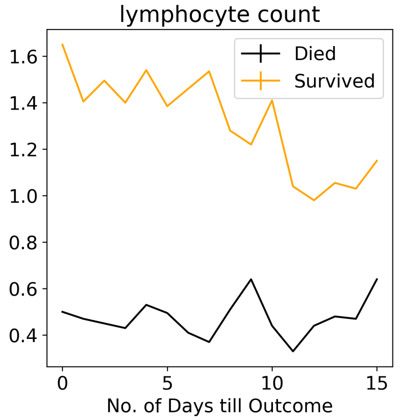

(i) Lymphocyte Count

Figure S18. Potential Features to predict number of days left to outcome: The median of features is determined on the set of data-points having the same number of days to outcome.
